# regQTLs: Single nucleotide polymorphisms that modulate microRNA regulation of gene expression in tumors

**DOI:** 10.1371/journal.pgen.1007837

**Published:** 2018-12-17

**Authors:** Gary Wilk, Rosemary Braun

**Affiliations:** 1 Department of Chemical and Biological Engineering, Northwestern University, Evanston, Illinois, United States of America; 2 Biostatistics Division, Feinberg School of Medicine, Northwestern University, Chicago, Illinois, United States of America; 3 Department of Engineering Sciences and Applied Mathematics, Northwestern University, Evanston, Illinois, United States of America; Beth Israel Deaconess Medical Center, UNITED STATES

## Abstract

Genome-wide association studies (GWAS) have identified single nucleotide polymorphisms (SNPs) associated with trait diversity and disease susceptibility, yet their functional properties often remain unclear. It has been hypothesized that SNPs in microRNA binding sites may disrupt gene regulation by microRNAs (miRNAs), short non-coding RNAs that bind to mRNA and downregulate the target gene. While several studies have predicted the location of SNPs in miRNA binding sites, to date there has been no comprehensive analysis of their impact on miRNA regulation. Here we investigate the functional properties of genetic variants and their effects on miRNA regulation of gene expression in cancer. Our analysis is motivated by the hypothesis that distinct alleles may cause differential binding (from miRNAs to mRNAs or from transcription factors to DNA) and change the expression of genes. We previously identified pathways—systems of genes conferring specific cell functions—that are dysregulated by miRNAs in cancer, by comparing miRNA–pathway associations between healthy and tumor tissue. We draw on these results as a starting point to assess whether SNPs on dysregulated pathways are responsible for miRNA dysregulation of individual genes in tumors. Using an integrative regression analysis that incorporates miRNA expression, mRNA expression, and SNP genotype data, we identify functional SNPs that we term “regulatory QTLs (regQTLs)”: loci whose alleles impact the regulation of genes by miRNAs. We apply the method to breast, liver, lung, and prostate cancer data from The Cancer Genome Atlas, and provide a tool to explore the findings.

## Author summary

Genomics studies have identified single nucleotide polymorphisms (SNPs) associated with trait diversity and disease susceptibility, yet the mechanism of action of many genetic variants remains unclear. MicroRNAs (miRNAs) are a class of small non-coding RNA molecules that base-pair coding mRNAs to regulate gene transcription. We hypothesize that SNP variants may affect the ability of miRNAs to bind their target genes, thus influencing gene regulation. To identify these “regulatory QTLs” (regQTLs), we integrate miRNA expression, mRNA expression, and SNP data to identify miRNAs that are associated with pathway dysregulation in tumors, and assess whether SNPs on these pathways are responsible for disrupted miRNA-gene regulation. This data-driven approach enables the discovery of SNPs whose alleles impact gene regulation by miRNAs, with functional consequences for tumor biology. We detail the method, apply it to data from The Cancer Genome Atlas, and provide a tool to explore the findings.

## Introduction

MicroRNAs (miRNAs) are small noncoding RNA molecules that modulate gene expression post-transcriptionally by means of complementary base pairing with mRNA transcripts. Through recognition of short target motifs (6-8 bases long) on the target mRNA, miRNAs bind and down-regulate the expression of the targeted gene. Regions flanking the “seed region” of the miRNA typically also bind the mRNA, creating a stronger annealing between the two RNA molecules. This results in the transcript being prevented from being translated into protein or degraded in the cell [1]. Because these molecular interactions are executed through base pairing, they can be influenced by genetic variation; changes in genome sequence may influence binding energy and the strength of annealing, or may even abrogate miRNA target sites entirely [2].

Polymorphisms constitute approximately 1% of the human genome and contribute to phenotypic diversity and susceptibility to disease. As such, large-scale resources to annotate known single nucleotide polymorphisms (SNPs) have been constructed, including dbSNP [3] and the International Hapmap Project [4], to describe patterns of genetic variation. Polymorphisms in miRNA and target site sequences have been implicated in aberrant miRNA-mRNA interactions and have been associated with multiple cancers [5–7], suggesting a link between genetic variation, miRNA regulation, and disease. Typically, discoveries of prognostic SNPs come from genome-wide association studies (GWAS), which statistically link variants with phenotypic traits. Recent GWAS studies have demonstrated that polymorphisms in miRNA binding sites increase the risk of breast [8, 9], bladder [10], and colon [11, 12] cancers, among others. In addition, several studies [2, 5] have suggested that polymorphisms within miRNA regulatory networks affect clinical outcomes and treatment responses.

In recent years, SNPs and their functional effects on miRNA regulation of genes have gained significant interest due to observed genetic variation within miRNA networks, and several databases and computational tools have been developed dedicated toward the study of polymorphic miRNA binding sites. These resources include PolymiRTS [13] (a database which links polymorphisms with miRNAs and target sites, in addition to diseases and biological pathways), Patrocles [14] (polymorphisms which are predicted to perturb miRNA-gene regulation, including eQTLs and Copy Number Variations), and dbSMR [15] (SNPs around miRNA target sites, genome-wide). These resources have improved the search for polymorphic binding sites and their potential functional effects in the cell. Analogous resources exist to study variation within transcription factors (TFs) and TF binding sites [16].

GWAS arrays are not comprehensive, however, and often under-sample genomic variants within known miRNA binding regions in the genome [17]. Additionally, while SNP variants may be predicted to affect miRNA-gene regulation based on their genomic position, the magnitude of the effect is often unclear. Hence, GWAS data alone is often insufficient to fully explore the relationship between genetic variation and miRNA regulation. Recently, researchers have combined GWAS data with separate miRNA expression data in head and neck squamous cell carcinoma to assess variants genome-wide affecting miRNA pathways in cancer [18]. There, the authors first conducted a GWAS to identify HSNCC-associated SNP loci, cross-referenced them against putative miRNA:mRNA binding sites, and confirmed that those miRNAs exhibited differential expression in the TCGA HSNCC data. To date, however, no attempts have been made to directly integrate SNP, miRNA, and gene expression data from the same samples to identify SNPs that disrupt miRNA–gene associations, and the functional effects of many polymorphisms and their molecular interactions remain unknown.

To consider the functional effects of SNPs in miRNA networks, several criteria are required as outlined in [19]. These criteria include independent association with the phenotype of interest, gene expression within the tissue, allelic changes which result in differential binding between miRNA and target gene(s), and resultant differential target gene expression. Concrete guidelines were suggested for future investigations to combine genetic and functional evidence for polymorphisms in miRNA target sites and human disease [7]. Follow-up functional experiments were suggested, in order to strengthen evidence of differential regulation. However, functional binding experiments are experimentally costly at scale, and are typically applied to specific systems of interest. As an alternative, several *in silico* tools have been developed to predict SNP effects on miRNA-gene interactions [20, 21]. However, these tools often fail to predict interactions that have been been observed in experiment [22].

To date, the functional effects of polymorphisms are typically explored by integrating GWAS and gene expression data find expression Quantitative Trait Loci (eQTLs): SNP variants that result in altered gene expression. Many eQTLs have been identified, including several associated with cancer. Recent integrative analyses using data from The Cancer Genome Atlas (TCGA) identified eQTLs in Breast Cancer [23] and Glioblastoma Multiforme [24, 25]. In fact, combinations of GWAS data with eQTL studies have found alleles that affect gene expression and complex traits genome-wide [26]. However, these analyses do not necessarily reveal the functional effects of polymorphisms on molecular-molecular interactions, particularly with respect to differential binding, as in miRNA-gene or TF-gene interactions.

Data from the TCGA project permits us to investigate the function of genetic variants by integrating SNP, gene expression, and miRNA expression from the same set of samples. Here, we propose a method to integrate these data to reveal genetic variants that show evidence of impacting miRNA-gene regulatory relationships. Motivated by the observation that integrative omics analyses provide more insight than single-platform approaches [27, 28], we perform an integrative omics analysis that searches for polymorphisms that modulate co-expression between miRNAs and their putative gene targets, which we term “regulatory QTLs (regQTLs)”: loci whose alleles impact the regulation of genes by miRNAs. Using mRNA expression, miRNA expression, and genotype data taken from tumor tissues, our method applies a regression model to assess whether disparate alleles present at a genomic variant modulate the miRNA-gene co-regulatory relationship. By comparing miRNA expression and gene expression across genotypes, we can identify regQTLs, or polymorphic sites which may alter molecular interactions and may be implicated in tumorigenesis. Importantly, by using miRNA and gene expression data, we avoid the inaccuracies associated with miRNA binding prediction algorithms, and are able to directly estimate the magnitude of the impact that the SNP has on the regulatory relationship.

Below, we present the method and apply it to TCGA data in Breast, Liver, Lung, and Prostate cancers. We report findings of gene variants that modulate miRNA regulation of gene expression in each of the cancer types studied. Interestingly, some of the flagged miRNAs and genes have been previously implicated in tumorigenic processes in the literature, and SNPs demonstrate functional changes to gene regulation. These results may have implications for future research in genomic regulation in tumors.

## Results

We identify regQTLs, genomic variants that influence miRNA regulation of gene expression, by integrating genomic and expression data from TCGA data. Specifically, we test whether different alleles at a SNP locus within a given gene alter how a miRNA modulates the expression of that gene across TCGA tumor samples. regQTLs may then provide context to gene regulation in cancer, due to genetic diversity or genetic alterations.

Previously [29], we had identified sets of genes, or pathways, whose overall activity appeared to be dysregulated by miRNAs in tumors in comparison to healthy tissue in four separate cancer types (breast, lung, liver, prostate). Our method first obtained an expression-based summary of pathway activity using Isomap [30], and then searched for differential miRNA correlations with the pathway summary across phenotypes, to find miRNA-pathway relationships at the systems level that were disrupted in cancer. Using data from The Cancer Genome Atlas (TCGA), we tested ∼10^5^ unique miRNA-pathway relationships, many of which were significantly dyregulated.

**Fig 1:**
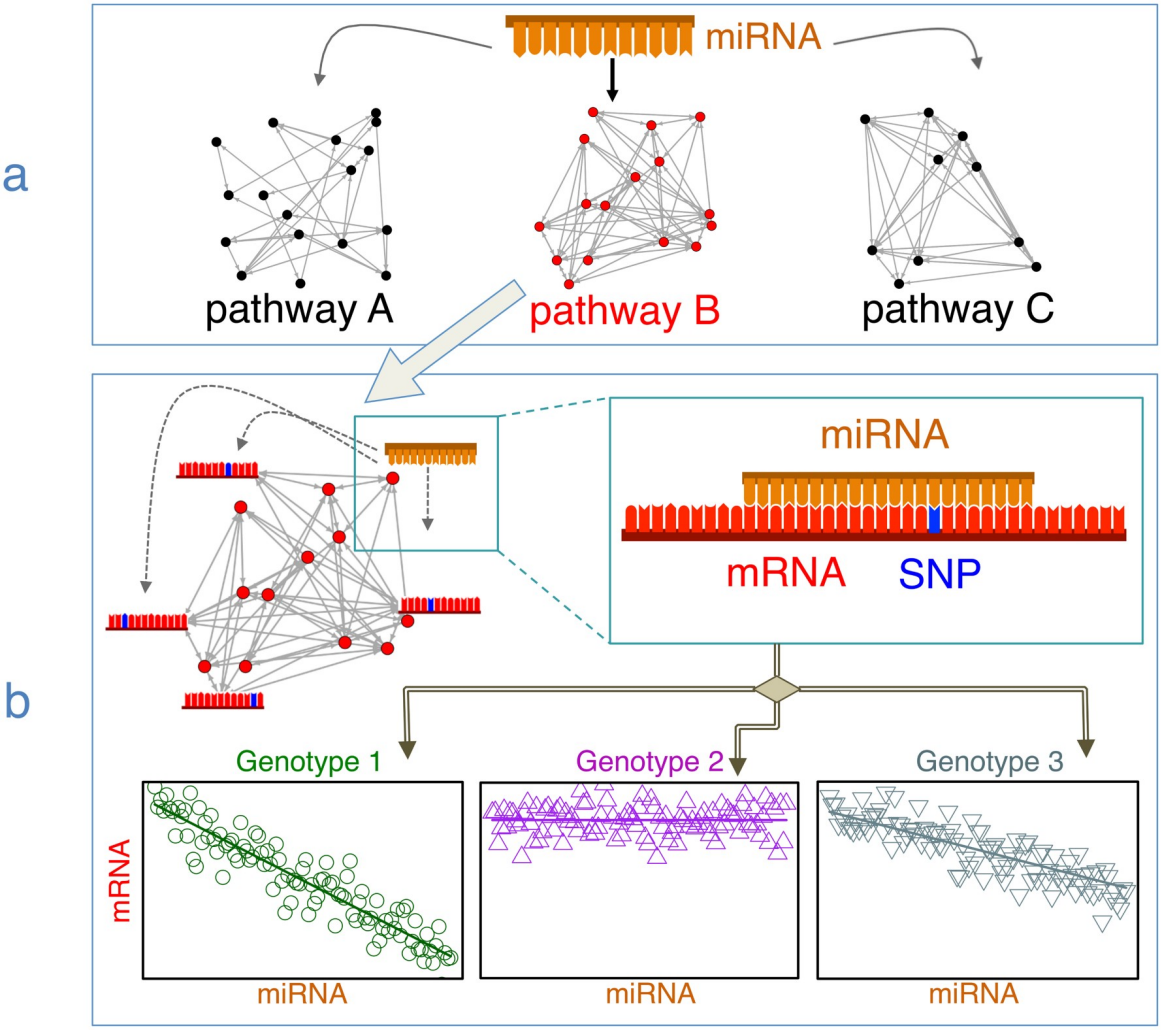
Illustration of the procedure to identify regQTLs. regQTLs: SNPs that modify the miRNA-mRNA relationship in dysregulated pathways. The method integrates gene expression, miRNA expression, and SNP data. (a) To aid mechanistic interpretability and reduce the search space, we first identify miRNA–pathway pairs that exhibit significant evidence of differential regulation following [29]. (b) Within each miRNA–identified pathway pair, we construct all miRNA-mRNA-SNP trios for each gene in the pathway (top), and systematically test whether the SNP modifies the expression relationship between the miRNA and the mRNA (bottom). Table 1 details the method.

Here we focus on those dysregulated miRNA-pathway pairs, and explore whether SNPs on the pathways are responsible for miRNA dysregulation of individual genes within that pathway. In other words, for each miRNA-pathway pair, we explore the co-expression patterns between the miRNA and the genes on the pathway, modulated by each of the polymorphisms located on the gene. By restricting our focus to genes in dysregulated miRNA-pathway pairs, we can ensure that the polymorphisms under consideration reside within perturbed systems in cancer. In addition, this restriction effectively reduces the dimensionality of our genome-wide analysis. We apply our methodology to TCGA data to explore all miRNA-mRNA-SNP combinations from miRNA-gene pairs where the gene was part of a dysregulated miRNA-pathway system, amounting to ∼10^6^ models per cancer type (breast, lung, liver, prostate). For each cancer, we report regQTLs which appear to modulate the co-regulatory miRNA-gene relationship in tumors and may therefore contribute to tumorigenesis. Fig 1 illustrates the intuition underlying the method.

### Detecting regQTLs

The steps of the method for detecting regQTLs are summarized in Table 1. Briefly, we consider all miRNA-gene pairs from dysregulated pathways that exhibited a differential association *p* < 0.01 in our prior analysis [29]. We systematically probe all unique miRNA-mRNA-SNP trios across all tumor samples in a cancer cohort. For each unique trio, we compute a multiple linear regression to model the expression of a gene as a response as a function of the miRNA expression, the SNP allele, and the interaction between them. We also adjust for population substructure. SNPs with strong interaction effects are inferred to be potential regQTLs. Full details of the analysis may be found in the Materials and Methods section.

**Table 1:** Procedure for assessing genomic variants modulating miRNA-gene interactions.

Method for finding regQTLs
Select dysregulated miRNA-pathway pairs (*p* < 0.01) following the method from [29] (Fig 1a). For each miRNA-pathway pair, find all genes on the pathway and all assayed SNPs on each gene to construct all unique miRNA-mRNA-SNP trios (Fig 1b, top). For each trio in Step 2, fit Eq 1 and apply ANOVA to assess statistical significance of the interaction terms (Fig 1b, bottom). FDR-adjust the resulting ANOVA *p*-values. Report highly significant miRNA-mRNA-SNP trios as potential regQTLs.

### regQTLs identified from TCGA data

**Fig 2:**
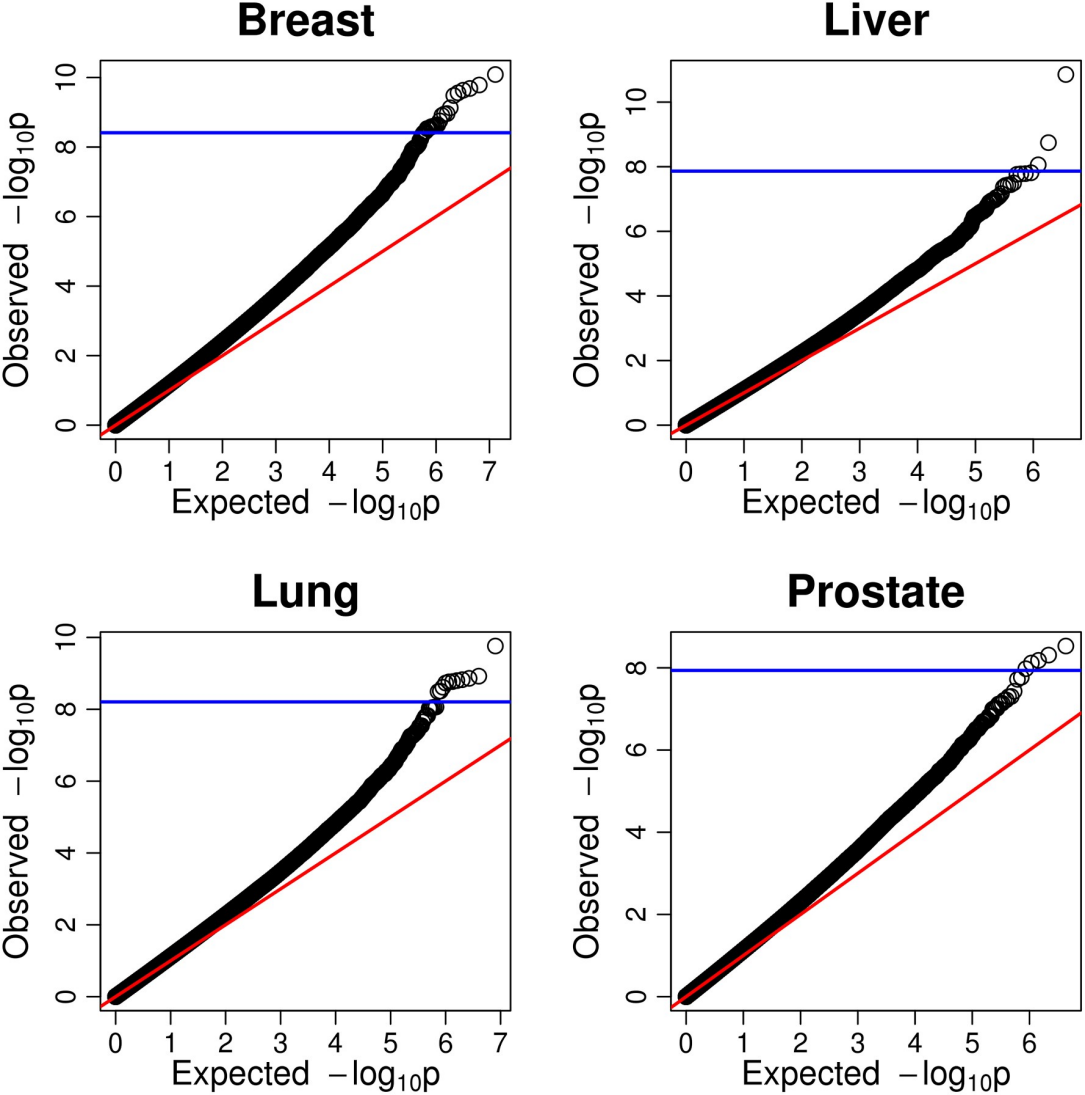
Quantile-quantile plots of regQTL interaction *p*-values. Quantile quantile plots of the observed *p*-values for the gene-miRNA-SNP ANOVA interaction tests versus their expected *p*-value distributions (the uniform distribution), tested in each cancer type. There were approximately 1.29 × 10^7^ unique interactions tested in breast, 3.65 × 10^6^ in liver, 8.03 × 10^6^ in lung, and 4.32 × 10^6^ in prostate cancer. A horizontal blue line indicates the threshold for genome–wide significance under the conservative Bonferroni adjustment. See also S1 Text.

We begin by presenting *qq*-plots of the regQTL *p*-values across all miRNA-mRNA-SNP trios in TCGA breast, liver, lung, and prostate cancer samples (Fig 2). It can be seen here that several trios in each study achieve extremely small *p*-values of *p* ≤ 10^−9^, indicating regQTLs that achieve genome–wide significance (even using the conservative Bonferroni correction).

It may also be observed that the distribution of regQTL *p*-values exhibit systematic deviations from the expected uniform distribution of *p*-values under the null, with many more significant observations than expected by chance for independent tests (as demonstrated by the trend away from the red diagonal lines). Such systematic deviations suggest that the trios are not strictly independent of one another; in classical GWAS, this is often attributable to population substructure driving the results. Here, however, some dependency amongst the tests is expected. Because we consider all known SNPs on each gene, many of the SNPs will be in linkage disequilibrium (LD) owing to their genomic proximity and will be correlated. Variants in LD have been observed in blocks ranging from tens of Kbp to greater than 100 Kbp [31], which may be larger than the size of a gene. In addition, because we consider genes within pathways, the expression of the genes may be correlated due to similar co-regulatory mechanisms or cooperative effects within a network. Because we expect the tests to exhibit some dependence, we perform multiple hypothesis adjustment using FDR [32, 33], rather than using the Bonferroni adjustment, which assumes independent tests and can be excessively conservative otherwise.

We note that population substructure may also be a factor in data drawn from diverse genetic populations. We tested for substructure by applying PCA to genotype data [34], and found that the first two principal components largely explained stratification by ethnicity/ancestry, as shown in S1, S2, S3 and S4 Figs. All results have been adjusted for population substructure by including the first two PCs in the models.

#### Breast cancer

**Fig 3:**
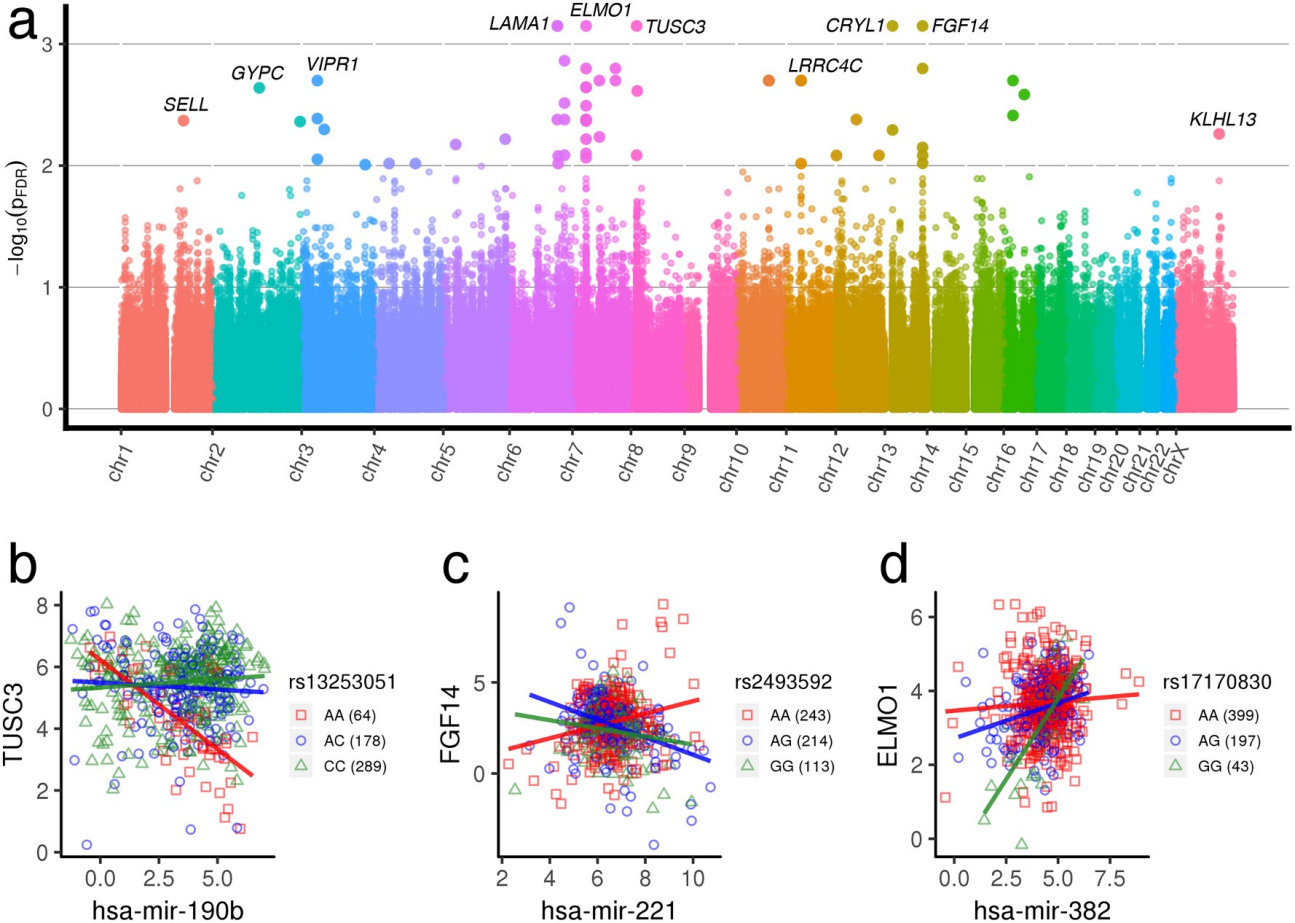
Breast cancer regQTLs. (a) Manhattan plot of regQTL −*log*_10_ *FDR* values in TCGA BRCA data. All gene-miRNA-SNP interaction*p*-values mapped to the location of the SNP in the genome. Observations are colored by chromosome. *p*-values are adjusted for the False Discovery Rate. A selection of strongly differentially regulated genes have been highlighted; others may be found using the mirSNP Shiny app or in the full data tables online. (b, c, d) Examples of breast cancer gene-miRNA-SNP trios with significant regulatory differences across genotypes (bottom). All trio interactions *p*_*FDR*_ < 0.005. More trios are indicated in S1 Text.

In breast cancer, ∼1.28 × 10^7^ unique gene-miRNA-SNP trios, drawn from 25,850 miRNA × pathway pairs, were analyzed and shown in Fig 3. Several chromosomes contain clusters of significant observations, as demonstrated by upward spikes within specific genomic regions. These clusters are composed of SNPs at different loci in close proximity with one another whose alleles are in LD. SNPs in LD are influenced by rates of recombination and mutation and reflect evolutionary history. Because of their genomic proximity, SNPs in linkage often lie within the same gene, such that multiple variants in a gene may wield similar biological effects on miRNA regulation, as demonstrated in Fig 3a.

This is evident when we observe regQTLs, all trios that were flagged as significant after FDR-adjustment (*FDR* < 0.1), of which relatively few achieve significance (2631). Among the flagged regQTLs, some miRNA-gene pairs are represented frequently, with multiple SNPs in the gene appearing to modulate the miRNA-gene relationship. Interestingly, of the miRNA–mRNA pairs identified within the significant trios, approximately 58% are predicted to have miRNA–mRNA target relationships according to TargetScan [35], with 68 actual validated miRNA–mRNA pairs according to mirTarBase [36]. S4 Table lists the miRNA-gene pairs with the largest number of significant regQTLs achieving significance with *FDR* < 0.1. (Note that S4 Table is not an exhaustive list but rather displays the highest number of modulating SNPs within miRNA-gene interactions at a given FDR).

It is notable that in several pairs in S4 Table, the miRNA is not predicted to target the gene based on sequence matching from [37]. This may be due to several factors. First, not all miRNA-gene interactions are known, and some interactions have been observed in experiment that have not been predicted through sequence matching [22]. Because these miRNA-gene pairs are modulated by many gene variants, these may represent novel biological interactions between miRNAs and genes that have yet to be documented and that are sensitive to biological variation. Another possibility is that these miRNAs and genes may not interact directly, but may be indirectly connected through second-order effects—for instance, one can envision a miRNA *target* interacting with the gene listed in the pair in S4 Table. This may lead to an apparent association with the miRNA, although it is mediated through another gene.

Selected examples of significant trios are shown in Fig 3b, 3c and 3d. For instance, in Fig 3b, samples with the homozygous minor (AA) genotype exhibit a strong negative dependence between hsa-mir-190b and *TUSC3*, whereas the heterozygous (AC) and homozygous major (CC) genotypes exhibit weaker and no dependencies. One explanation may be that samples having both A alleles confer strong binding between hsa-mir-190b and *TUSC3*, whereas the introduction of the C allele confers weaker (AC) or no binding (CC) at all. hsa-mir-190b is predicted to target *TUSC3* by sequence matching, and both the miRNA and gene are implicated in cancer in the literature. *TUSC3* is a tumor suppressor whose loss or decreased expression is associated with the proliferation of several cancer types [38–40] and is markedly under-expressed in breast cancer cells [41]. hsa-mir-190b has recently been found to be the most upregulated miRNA in ER*α* breast cancers relative to ER*α* negative breast cancers [42], and is part of the regulatory network that activates *TP53* [43]. Likewise, in Fig 3c, hsa-mir-221 is predicted to target *FGF14* and exhibits regulatory differences across genotypes. In this case, the homozygous minor (AA) appears to confer a loss of regulation, whereas the introduction of the G allele in the heterozygous (AG) and homozygous major (GG) genotypes, confers negative regulation and perhaps strong binding. hsa-mir-221/222 has previously been associated with a basal-like phenotype and the epithelial to mesenchymal transition in breast cancer [44]. Although *FGF14* in particular is not implicated in cancer, aberrant signaling of other Fibroblast Growth Factors are widely found in the pathogenesis of cancer [45].

In Fig 3d, the anomalous genotype, homozygous major (GG), is associated with a strong positive dependence between hsa-mir-382 and *ELMO1*, in contrast to the other panels. hsa-mir-382 is predicted to target *ELMO1* according to TargetScan [35]. Altered binding of miRNA–mRNA pairs due to genetic variation or mutations may promote sequestration of the mRNA, and therefore generate positive dependence between miRNA and mRNA expression. Alternatively, we may flag second-order effects for positive dependence between miRNA-mRNA pairs by genotype. *ELMO1* is associated with metastasis in several cancers, and is part of the chomokine regulated pathway, including Rac1 and Rac2, that regulates the actin cytoskeleton in metastatic breast cancer [46]. hsa-mir-382 has been found to promote breast cancer invasion and metastasis by activating the Ras/ERK pathway in breast cancer cells [47]. The examples shown in Fig 3 are illustrative of the types of regulatory interactions affected by genomic variation we observe within breast cancer.

Breast cancer in particular appears to exhibit rather large heterogeneity in comparison to other cancer types (Fig 2). Because breast cancer patients may be susceptible to different outcomes and clinical approaches based on receptor status, we include subset analyses for breast tumors with ER+, PR+, and triple negative breast cancer in the Supporting information. In brief, each receptor subtype exhibits more significant interactions than expected by chance, as does breast cancer in aggregate. In addition, regQTL trios differ in interaction significance by subtype, which may have relevance for clinical treatment.

#### Liver cancer

**Fig 4:**
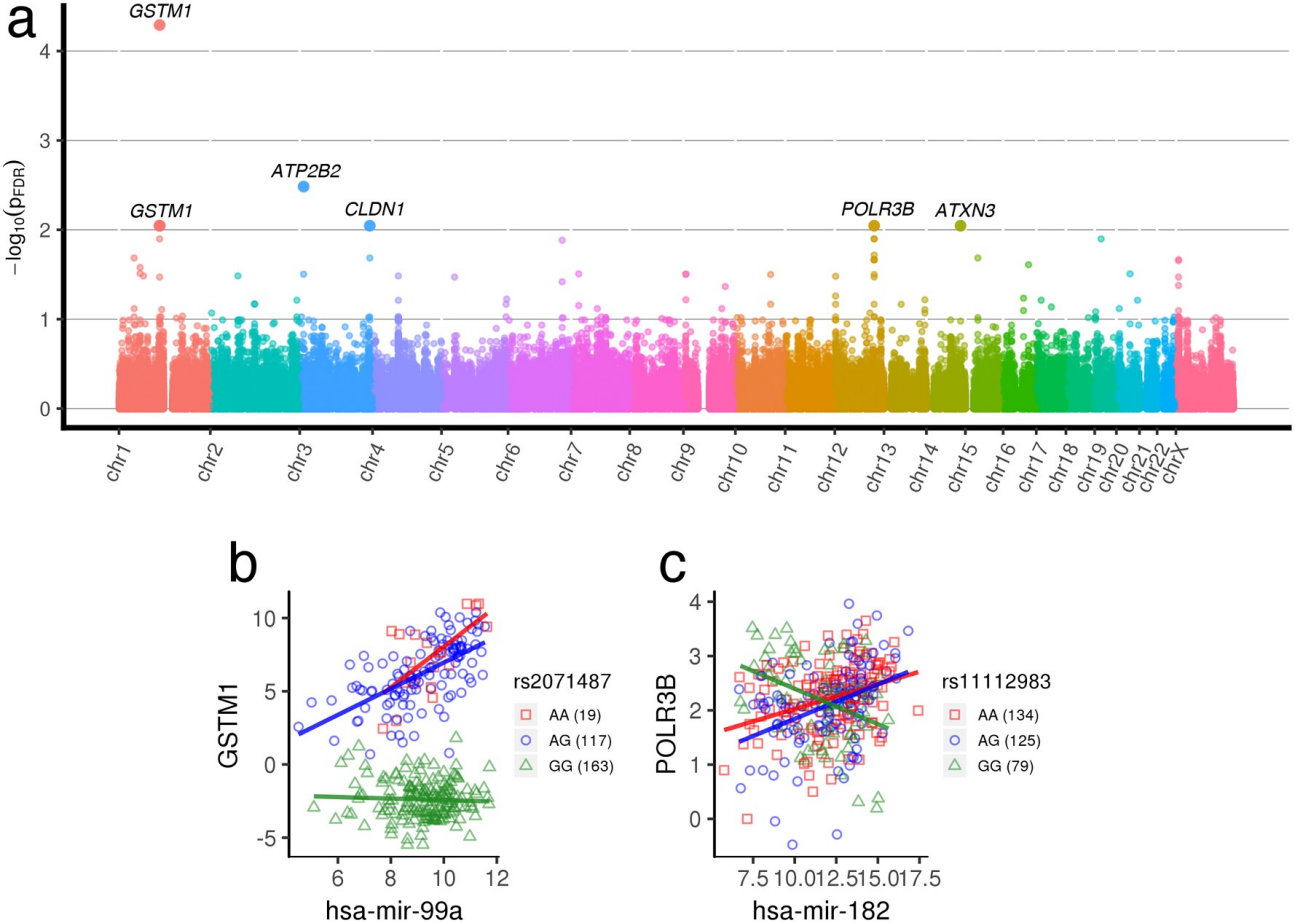
Liver cancer regQTLs. (a) Manhattan plot of regQTL −*log*_10_ *FDR* values in TCGA LIHC data. (b, c) Examples of liver cancer gene-miRNA-SNP trios with significant regulatory differences across genotypes. All trio interactions *p*_*FDR*_ < 0.005. More trios are indicated in S1 Text.

A total of 3.65 × 10^6^ gene-miRNA-SNP unique trios, drawn from 7371 miRNA × pathway pairs, were mapped to their loci in the genome in Fig 4 for liver cancer. Although fewer trios achieve significance in comparison to breast cancer (108 regQTLs with *FDR* < 0.1), we again observe the associated SNPs to be clustered due to linkage (Fig 4a). Furthermore, approximately 40% of the miRNA–mRNA pairs in significant trios have predicted miRNA–mRNA relationships from TargetScan [35] with only three validated miRNA–mRNA pairs from mirTarBase [36]. We illustrate a noteworthy genotype-dependent interaction in Fig 4b. Not only is *GSTM1* differentially regulated by hsa-mir-99a at rs2071487 depending on genotype, but also *GSTM1* exhibits initial genotype-dependent gene expression differences typical of a strong eQTL. In this case, alleles appear to have the power to determine both expression and modulation of gene regulation. *GSTM1* is part of the GST-superfamily that detoxifies electrophilic compounds by conjugation with glutathione, and is involved in processing carcinogens, drugs, and toxins. *GSMT1* is highly polymorphic, affecting toxicity and drug efficacy across individuals, and in particular, null mutations are associated with an increase in susceptibility to lung, bladder, and colon cancers [48]. hsa-mir-99a inhibits hepatocellular carcinoma growth [49] and its dysregulation is an early marker of tumor progression [50]. While hsa-mir-99a is not predicted to target *GSTM1*, it is predicted to target *GSTM3* and *GSTM5*, other GST-superfamily *μ* enzymes.

S5 Table presents the miRNA-gene pairs that have the greatest number of regQTLs. The top gene, *POLR3B*, contains 6 SNPs that each modulate its regulation by hsa-mir-182, depending on genotype. We illustrate one such example in Fig 4c, in which the anomalous genotype (GG) for rs11112983 downregulates expression of *POLR3B* by hsa-mir-182, in comparison to the others. *POLR3B* is subunit B (the second largest) of RNA polymerase III, which is the polymerase that synthesizes transfer and small ribosomal RNAs. Increased RNA polymerase III output is widely implicated in cancer [51]. Recently, a novel truncated version of *POLR3B* called *INMAP* has been observed to repress AP-1 and *TP53* activity and is upregulated in several cancer cell lines [52]. hsa-mir-182 is significantly upregulated in hepatocellular carcinoma and has been found to promote proliferation and invasion by downregulating tumor suppressor *EFNA5* [53] and promote metastasis by downregulating metastasis suppressor 1 [54]. While hsa-mir-182 itself isn’t predicted to target *POLR3B*, it is predicted to target other subunits on RNA polymerase III, and therefore may exert second-order regulatory effects with *POLR3B*.

#### Lung cancer

**Fig 5:**
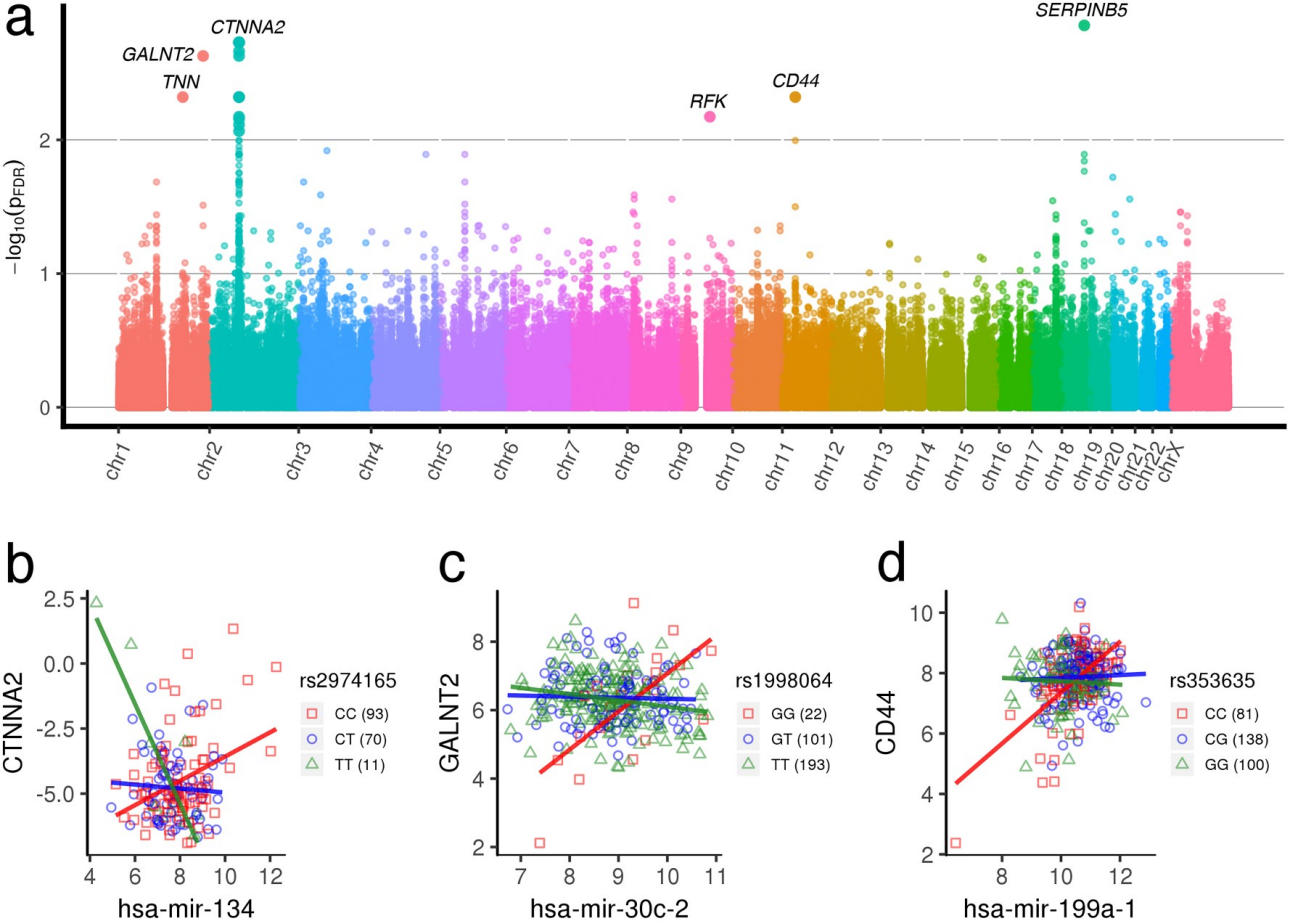
Lung cancer regQTLs. (a) Manhattan plot of regQTL −*log*_10_ *FDR* values in TCGA LUSC data. (b, c, d) Examples of lung cancer gene-miRNA-SNP trio with a significant regulatory difference across genotypes. All trio interactions *p*_*FDR*_ < 0.015. More trios are indicated in S1 Text.

A total of 8.03 × 10^6^ unique gene-miRNA-SNP trios, drawn from from 14433 miRNA × pathway pairs in lung cance were mapped to their loci in the genome in Fig 5. Of the miRNA–mRNA pairs identified in the 338 significant trios, approximately 64% are predicted to have miRNA–mRNA target relationships according to TargetScan [35], with 10 experimentally validated miRNA–mRNA pairs from mirTarBase [36]. Among the clusters of correlated observations that spike in Fig 5a, several miRNA-gene pairs are represented frequently and tabulated in S6 Table. *COL11A1*, *MAOA*, and *CTNNA2* on Chromosomes 1, X, and 2, respectively, collectively make up the bulk of the genes containing SNPs modulating their observations in LD with each other.

In Fig 5b, rs2974165 modulates the association between *CTNNA2* and hsa-mir-134, with strong negative regulation for the homozygous minor (TT) genotype. *CTNNA2* and *CTNNA3* encode for cell-cell adhesions, and are thought to be tumor suppressors which are frequently mutated and implicated in head and neck squamous cell carcinoma [55]. Additionally, *CTNNA2* has been thought to have an important role in cell migration and metastasis [56]. hsa-mir-134 is not predicted to target *CTNNA2*, but rather *CTNNA3* in TargetScan [35]. hsa-mir-134 is a tumor suppressor miRNA that is downregulated in many cancers, including head and neck cancer [57]. Furthermore, hsa-mir-134 regulates proliferation, invasion, and metasasis in lung tissue, and is underexpressed in non-small cell lung cancer [58].

Yet another example of anomalous regulation we detect is shown in Fig 5c. Here the homozygous major genotype (GG) exhibits a strong positive correlation between hsa-mir-30c-2 and *GALNT2*, in contrast to the other genotypes, which exhibit no appreciable correlation. hsa-mir-30c-2 is predicted to target *GALNT2* in TargetScan [35], indicating that we may be detecting allele-specific differences on miRNA regulation of genes, or endogenous sequestration of the mRNA due to altered binding. *GALNT2* is an O-glycosylating enzyme that regulates the EGFR receptor. *GALNT2* has been demonstrated to suppress malignancy in several cancers, and its dysregulation has been implicated in tumor progression [59, 60]. Likewise, hsa-mir-30c-2 has been found to be a regulator of signal transduction and cell cycle progression in ovarian [61] and breast cancer [62] cells.

Another example of anomalous regulation we detect is shown in Fig 5d. Similar to Fig 5c, the homozygous major genotype (CC) exhibits a strong positive correlation between hsa-mir-199a-1 and *CD44* for rs343635, unlike the other genotypes which exhibit no appreciable correlation. *CD44* is a lymphocyte hyaluronan receptor that has been implicated as a marker in cancer stem cells, and contributes to their metastasis in the tumor microenvironment [63, 64]. hsa-mir-199a-1 and its parent miRNA hsa-mir-199a is downregulated in hypoxia environments and has been reported to have tumor suppressor properties in several cancer types [65–67].

We note that in S6 Table, *CTNNA2* appears frequently with several miRNAs, often with the same SNPs. Because the anomalous SNPs in *CTNNA2* have relatively low representation among the cohort, it is difficult to attribute confidence to their regulatory effect. That is, genotype frequencies for the homozygous minor alleles (MAF: 28%–41%) may be undersampled for these SNPs (range: 9.9%–16.1%). Another note is that *CTNNA2* has low gene expression in lung tumor samples (< 0.1 TPM for ∼ 92% of the samples). This may subject it to biological fluctuations, due to noise and excluded samples, which may influence results.

#### Prostate cancer

**Fig 6:**
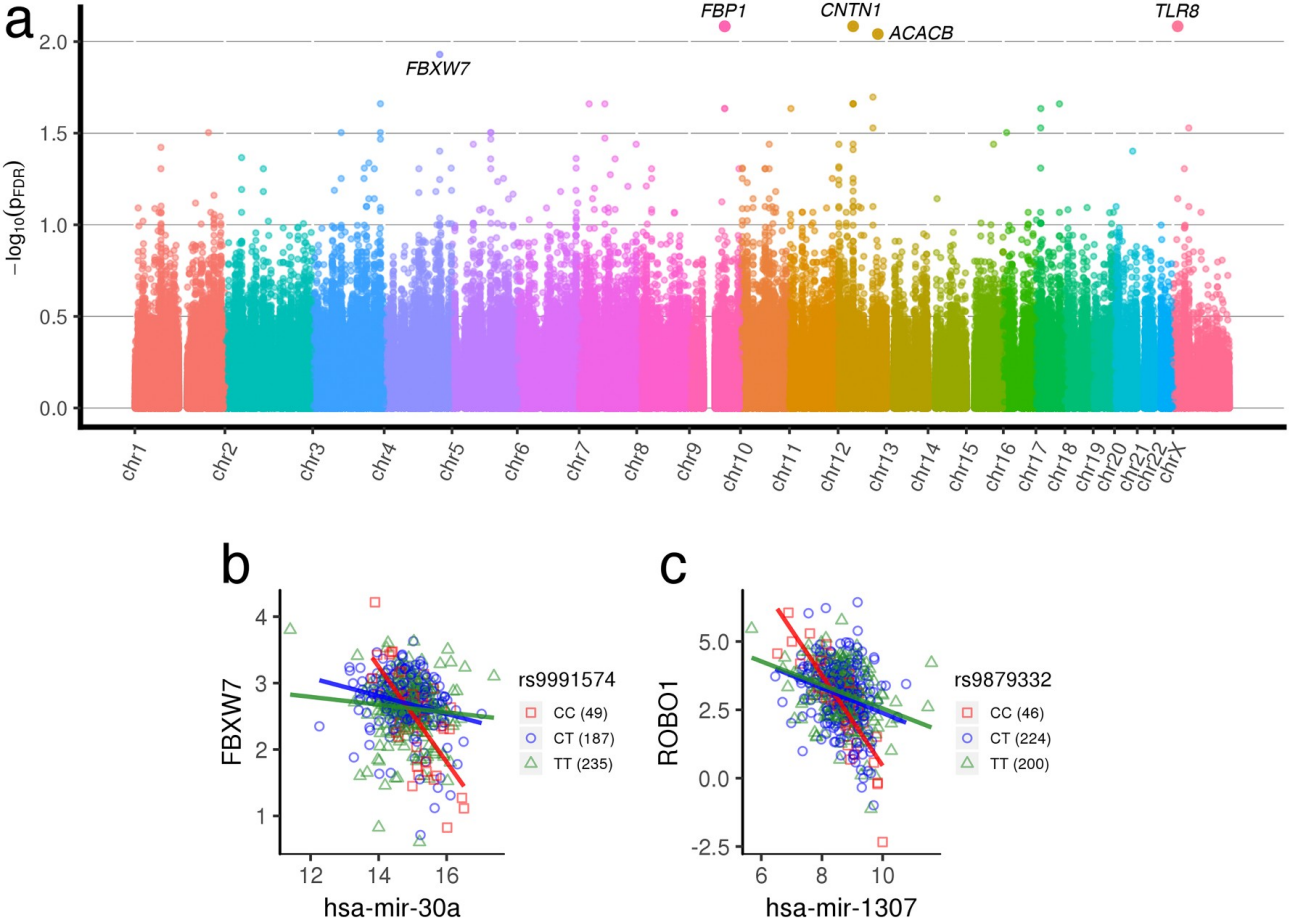
Prostate cancer regQTLs. (a) Manhattan plot of regQTL −*log*_10_ *FDR* values in TCGA PRAD data. (b, c) Examples of prostate cancer gene-miRNA-SNP trios with significant regulatory differences across genotypes. All trio interactions *p*_*FDR*_ < 0.05. More trios are indicated in S1 Text.

A total of 4.3 × 10^6^ unique gene-miRNA-SNP trios, drawn from 8673 miRNA × pathway pairs in prostate cancer were mapped to their loci in the genome in Fig 6. The scale in Fig 6a is lower than the Manhattan plots in the other cancer types, with the most significant interaction *p* ≈ 0.00825. Nevertheless, we do observe pairs containing many SNPs modulating their interactions in S7 Table. In addition, many of these miRNA-gene pairs are predicted to interact biologically based on sequence matching, and 66% of the miRNA–mRNA pairs in the 209 significant trios are predicted to have miRNA–mRNA target relationships according to TargetScan [35], of which 8 are experimentally validated [36].

A few examples of trios we find in prostate cancer are shown in Fig 6. In Fig 6b, hsa-mir-30a is predicted to target *FBXW7*, and shows a sharp negative regulatory dependence for the CC genotype, whereas the other genotypes exhibit no appreciable miRNA-gene regulation. hsa-mir-30a and *FBXW7* are among the most frequently flagged pairs we find in prostate cancer. hsa-mir-30a is a tumor suppressor that inhibits EMT genes that is typically down-regulated by oncogenic signals in prostate cancer like *EGF*, particularly in metastasis [68]. *FBXW7*, an F-box protein, mediates ubiquitination and proteasomal degradation of target proteins. Its down-expression, loss, and frequent mutation is shown in multiple cancer types, including ovarian, breast, melanoma, colon, and others. In Fig 6c, the CC genotype exhibits strong negative regulation between hsa-mir-1307 and *ROBO1*, whereas the other genotypes exhibit weaker dependencies. hsa-mir-1307 is predicted to target *ROBO1*, and promotes proliferation in prostate cancer by targeting *FOXO3A* [69]. *ROBO1* itself is part of the immonoglobulin gene superfamily and is an axon guidance receptor gene previously implicated in dyslexia.

### Tool for interactive exploration of complete results

We have presented only a few examples of gene-miRNA-SNP trios tested in the cancer types shown above. In order to enable researchers to explore other trios, we have produced an open source R Shiny application, called mirApp, that is freely available and can be used to investigate other trios in the analyzed datasets. The user is asked to choose the cancer type (breast, liver, lung, or prostate) and input a specific miRNA of interest. The Shiny app then produces a miRNA-specific Manhattan plot of all significant regQTLs within the cancer type (*p*_*FDR*_ < 0.1). This Manhattan plot is interactive, such that when the user clicks on an individual regQTL point, the app produces its trio interaction plot, similar to Fig 3b. mirApp can be downloaded freely from https://github.com/gawilk/mirApp.

## Discussion

We have described a novel integrative method that combines genomic and expression data to elucidate the effects that genomic variants exert on miRNA regulation of genes in cancer. This integrative analysis combines miRNA expression, mRNA expression, and genotype data from tumor tissue to find polymorphisms that modulate co-expression patterns between miRNAs and their putative gene targets, which we term regQTLs. This analysis continues our previous work that identified miRNAs and entire pathways whose co-regulation was found to be disrupted in tumors. Here, we hone in on previously identified dysregulated pathways, and determine whether polymorphisms present within pathway genes may contribute to individual gene dysregulation.

This work is in the spirit of other integrative omics analyses to yield insights into gene expression regulatory mechanisms. Its main novelty is to take into account genomic variation and apply it on a genome-wide scale. Other integrative analyses of omics platforms have been applied to yield discoveries on gene expression regulation mechanisms. Pipelines such as CrossHub [70] take into account miRNA and TF relationships as well as methylation evidence, through TCGA and ENCODE ChIP-Seq binding evidence, to describe regulation of gene expression. RACER [71] uses regression analysis to predict gene expression as a function of genetic and epigenetic factors including copy number variation, miRNAs, DNA methylation, and TF evidence combined from TCGA and ENCODE to study Acute Myeloid Leukemia. Another study [72] combined similar input variables in a linear fashion to model mRNA expression changes in glioblastoma tumor samples, and was able to identify activities that were predictive of subtypes and survival. These studies have identified some relationships between expression regulators and genes and focused on mostly single cancer types. Jacobsen and colleagues [73] used a statistical approach to model the recurrence of miRNA-mRNA expression in tumor samples across multiple cancer types, induced by changes in DNA copy number and promoter methylation. However, none of these studies have taken into account genomic variation to address their effect on gene regulation.

In contrast, our method incorporates genomic variation to identify regQTLs. Our method is fully data driven, integrating sample specific expression and genomic data to find allele-specific regulatory effects. By applying multiple linear regression models using all three omics features, we can assess which SNPs differentially affect miRNA regulation of genes. The use of linear models and ANOVA allow for relatively easy assessment of statistical interactions. Because we focus on genes within pathways found to be co-regulated with miRNAs which are disrupted in cancer, our approach may help find genomic variants that contribute to tumorigenesis. We emphasize that the application to dysregulated pathways permits the identification of regQTLs with potentially local and system effects, and significantly reduces the search space of mechanisms under consideration in the genome.

We apply this analysis to breast, liver, lung, and prostate cancers, and within each cancer type, test millions of possible models (genes regulated by miRNAs modulated by SNPs, or “trios”). We find polymorphisms systematically affecting miRNA-gene regulation, with many more statistically significant effects than expected by chance. This supports the notion that cancer contains significant perturbations to the entire genome. Among the flagged trios with high significance, many miRNAs and genes are often implicated in tumorigenic processes in the literature. These include tumor suppressor genes, genes in the the p53 network, genes within signaling pathways, and miRNAs whose aberrant expression or aberrant targeting has been documented in multiple cancer types. In addition, we find several genes each containing many individual variants differentially modulating miRNA regulation. These genes, and the genomic regions surrounding them, may indicate hotspots of tumorigenic interest for future research.

Due to the extensive nature of the study, an R Shiny app has been developed to fully explore all regQTLs and visualize their effects. Users can utilize the app interactively to observe regQTL significance genome-wide by cancer type, and plot individual miRNA-gene interactions modulated by them. This utility allows for complete exploration of our integrative analysis of TCGA data.

We note that the our results are somewhat limited by the input data. Currently, TCGA is the largest known resource of cancer omics data, with samples assayed across both expression and genotype data. However, individuals of European ancestry are highly overrepresented. Having comparable datasets in diverse populations would strengthen the results of this study. In addition, our method only considers genes and SNPs which lie on annotated pathways; genes and SNPs that are currently unannotated on biological pathways, and therefore unconsidered in our model, may be of tumorigenic importance. Furthermore, we utilized tag SNPs in our study, which are chosen as markers of genomic structure rather than because of their functional importance. Tag SNPs may not have obvious mechanistic roles, but are easily assayed and can serve as proxies for other nearby genomic variants in linkage disequilibrium. Thus, tag SNPs may permit the discovery of novel functional SNPs. Finally, our search for regQTLs is highly flexible; we do not restrict miRNA-gene relationships to those already corroborated with biophysical evidence, and, in addition, do not restrict genomic variants to those within putative miRNA binding regions. These criteria have been set to allow for novel discoveries, since computational miRNA-gene binding rules have been observed to deviate from experiment. However, we may also detect sequestration effects of mRNA transcripts due to altered binding, or second-order effects of miRNA regulation modulated by genotype (or possibly spurious relationships). Functional experiments, including reverse protein phase array (RPPA) data, may help elucidate the biological mechanism (if any) of these relationships. Nevertheless, we do find significant relationships in which the genes and miRNAs are implicated in cancers in the literature.

Our model is relatively simple and can be efficiently applied to any combined miRNA/mRNA/SNP dataset of interest to reveal the effects of a single regulatory SNP. We envision that future work could apply and extend our approach in several ways. For instance, it is conceivable that multiple SNPs in combination will influence miRNA regulation of a gene, and that genomic variation may affect other layers of gene regulation (e.g., but influencing transcription factor binding). Future extensions of this method could include integrating TF binding sites or epigenetic factors in the analysis regQTLs. Given specific regQTLs identified in this study, other avenues could include validating their differential regulation by experimental means, or estimating their strength *in silico*. Perhaps the most exciting future application would be to inform personalized medicine in the context of miRNA therapeutics [74–76]; for instance, the results shown in Fig 3b suggest that targeting hsa-mir-190b could influence the expression of the tumor suppressor *TUSC3*, but only amongst homozygous AA individuals at regQTL rs13253051.

Finally, we note that our method for identifying regQTLs can be easily applied to other diseases and experimental modalities (such as TFs) to determine the functional impact of specific loci. A genome-wide analysis of functional regulatory effects can help identify polymorphisms and mutations that contribute to disease.

## Materials and methods

### Analytical approach

We seek to identify SNPs whose alleles alter the relationship between the expression of a miRNA and gene. To this end, for a SNP with genotypes {*AA*, *Aa*, *aa*}, we fit the model

$$\begin{array}{c} \begin{array}{c} Y=\beta_{0}+\beta_{1}PC1+\beta_{2}PC2+\beta_{3}X_{\text{miR}}+\beta_{4}1\left(\text{SNP = Aa}\right)+\beta_{5}1\left(\text{SNP = aa}\right)+\\ \beta_{6}1\left(\text{SNP = Aa}\right)X_{\text{miR}}+\beta_{7}1\left(\text{SNP = aa}\right)X_{\text{miR}}+\varepsilon \;, \end{array} \end{array}$$ (1)

where *Y* represents the expression level of the gene of interest, *PC*1 and *PC*2 represent the first two principal components of the SNP genotypes to correct for population stratification, respectively, *X*_miR_ is the expression level of the miRNA, and 1(·) is an indicator function for the SNP genotype. In this model, the coefficients *β*_1_ and *β*_2_ quantify the effect of population structure on the observed gene expression; *β*_3_ quantifies the relationship between the miRNA and gene expression for the reference genotype *AA*; the coefficients *β*_4_, *β*_5_ quantify how the allele affects overall expression of the gene (i.e., as an eQTL); and the interaction coefficients *β*_6_, *β*_7_ quantify how the variant alleles at the SNP of interest modulate the miRNA-mRNA relationship. SNPs with strong interaction effects are inferred to be potential regQTLs.

SNPs are treated as categorical variables in our model to capture any dominant, recessive, or additive effects that individual alleles may confer on miRNA-gene interactions. Because any single copy of an allele may create, strengthen, weaken, or abrogate miRNA-gene binding, we seek to capture all possible SNP effects and their cross-comparisons. For instance, an allele that creates strong miRNA-gene binding may only need to be present in one copy to show an effect, such that the salient difference is observed between having no copy of the variant allele and having one or two copies (with no difference between one and two). Alternatively, an allele that abrogates miRNA-gene binding may be seen to have a strong effect for those with homozygous copies, but a much weaker effect for heterozygous individuals. As such, we explore all allelic effects on miRNA-gene binding.

To assess the statistical significance of the interaction effect, we apply ANOVA Type III sums of squares (Yates’s weighted squares of means) to compare the full model to that without the interaction terms. A significant *F* statistic for the interactions suggests that at least one of the variant SNP alleles substantially alters the relationship between the miRNA and the mRNA, on top of any eQTL-like effects. *p*-values for all interactions are then FDR-adjusted [32] for the large number of miRNA-mRNA-SNP trios probed in the dataset. (We choose this Benjamini-Hochberg FDR adjustment because we expect that models with common miRNAs or genes are not strictly independent, and this procedure has been show to provide control of the FDR under dependency [33]).

### Application to TCGA data

As a proof of concept, we applied this method systematically to tumor samples with combined miRNA expression, gene expression, and SNP genotype data from TCGA. Code to carry out the analysis (to reproduce our results or apply them to other SNP/miRNA/mRNA datasets), and full data tables, can be obtained from https://github.com/gawilk/miRNA-SNP.

#### Data

TCGA data were downloaded for BRCA (breast), LIHC (liver), LUSC (lung), and PRAD (prostate) cancers from https://portal.gdc.cancer.gov. Tumor samples (TCGA sample type “01”) measured across mRNA IlluminaHiSeq_RNASeqV2 (Level 3), miRNA IlluminaHiSeq_miRNASeq (Level 3), and Affymetrix SNP6.0 platforms were used for the analysis, amounting to 699 total tumor samples in breast, 345 in liver, 341 in lung, and 481 in prostate cancer.

#### Data preprocessing and filtration

Briefly, mRNA data were converted to TPM and log2 transformed, and miRNA data were log2 transformed (both with small offsets for the log transformation). In addition, genes and miRNAs were removed from consideration that had very low expression across most samples in the set (defined as genes with median expression < 10^−9^ before TPM conversion and miRNAs having expression ≤ 1 for more than half of the samples in the set before log transformation). In total, we considered 5869 pathway-associated genes from KEGG.

SNPs were filtered out that had a low Birdseed confidence threshold (0.05) for genotype calls in the TCGA pipeline. We used two additional filtration criteria to remove SNPs: a) those having minor allele frequencies (MAF) less than 1% and b) those having genotype frequencies less than 5% across all samples in a cancer dataset. These criteria were imposed to ensure that limited sampling of rare alleles and genotypes would not skew the regression results. In total, we considered 72901 unique SNPs in breast cancer, 70142 SNPs in liver cancer, 73037 SNPs in lung cancer, and 66967 SNPs in prostate cancer. Summary statistics for the genomic context of the SNPs may be seen in the Supporting information section. In each cancer type, the first two principal components largely explained stratification by ethnicity/ancestry; each remaining component explained < 1% of the variance. Thus, we used the first two principal components to account for population substructure in the regressions.

In our miRNA-pathway analysis [29], each cancer type exhibited a very unique regulatory pathology, with little to no miRNA-pathway overlap among cancers. Because miRNA-mRNA-SNP trios were selected from dysregulated miRNA-pathway pairs, we found no commonality of the trios among cancer types. Before applying the regression models, individual samples within a miRNA-mRNA-SNP trio having no appreciable miRNA expression were removed from consideration, since they are not biologically of interest. Additionally, samples having a large Cook’s Distance (*D* > 1) were removed from the regressions and the regressions were recomputed to limit the influence of outliers on the resulting models.

## Supporting information

S1 Text Supporting information for the main text. (PDF)

S1 Table Summary statistics for SNPs. Numbers denote unique SNPs within each genomic region per cancer type.
(PDF)

S2 Table Significant regQTLs (< 0.1 FDR) and their statistics. “Proportion” denotes the fraction of unique miRNA–mRNA relationships that are predicted by TargetScan [35], out of all unique miRNA–mRNA relationships found within the “total” number of significant regQTL trios. “Validated” denotes the unique number of experimentally validated miRNA–mRNA relationships according to mirTarBase [36], out of the significant regQTL trios.
(PDF)

S3 Table Global review of significant regQTLs (at < 0.1 FDR). Genes denotes the number of genes containing > 1 SNP modulating miRNA–mRNA interactions, and the number of tumor suppressors and/or oncogenes according to [77] in each cancer type.
(PDF)

S4 Table miRNA-gene pairs containing the greatest number of genetic variants significantly modulating their interactions in breast cancer. “SNPs” indicates the number of associated SNPs on the gene found to significantly modulate (at *p*_*FDR*_ < 0.1) the miRNA-gene interaction, out of the total number of known SNPs on the gene. *MAF*_*avg*_ indicates the average minor allele frequency of the SNPs located on the gene. *p*_*MIN*_ indicates the most significant interaction *p*-value after FDR-correction. “chr” indicates the chromosome where the SNP is located. “predicted” indicates whether the miRNA is predicted to target the gene based off sequence matching from microRNA.org. “pathways” indicates the number of KEGG pathways the gene is part of.
(PDF)

S5 Table miRNA-gene pairs containing the greatest number of genetic variants significantly modulating their interactions in liver cancer. (PDF)

S6 Table miRNA-gene pairs containing the greatest number of genetic variants significantly modulating their interactions in lung cancer. (PDF)

S7 Table miRNA-gene pairs containing the greatest number of genetic variants significantly modulating their interactions in lung cancer. (PDF)

S1 Fig PCA plot of breast SNP genotype data. The population is stratified by ancestry/ethnicity, and substructure is largely explained by the first two principal components.
(PDF)

S2 Fig PCA plot of liver SNP genotype data. (PDF)

S3 Fig PCA plot of lung SNP genotype data. (PDF)

S4 Fig PCA plot of prostate SNP genotype data. (PDF)

S5 Fig Proportion of variance explained by the first six principal components. (PDF)

S6 Fig Venn diagram of overlapping samples with ER+, PR+, and triple negative breast cancer. (PNG)

S7 Fig Quantile-quantile plots of breast cancer subtype regQTL interaction *p*-values. Quantile-quantile plots of the observed *p*-values for the gene-miRNA-SNP ANOVA interaction tests versus their expected *p*-value distributions (the uniform distribution), tested in ER+, PR+, and triple negative breast cancer subtypes.
(PNG)

S8 Fig Example trio plots in ER+ (left plot) and PR+ (right plot) breast cancer subtypes. (PDF)

S9 Fig Overlap of genes (left) and miRNAs (right) across cancer types in significant regQTL trios. (PDF)

## References

[1] Bartel DP. MicroRNAs: genomics, biogenesis, mechanism, and function. Cell. 2004;116(2):281–297. 14744438 10.1016/s0092-8674(04)00045-5

[2] Chen K, Song F, Calin GA, Wei Q, Hao X, Zhang W. Polymorphisms in microRNA targets: a gold mine for molecular epidemiology. Carcinogenesis. 2008;29(7):1306–1311. 10.1093/carcin/bgn116 18477647

[3] Sherry ST, Ward MH, Kholodov M, Baker J, Phan L, Smigielski EM, et al dbSNP: the NCBI database of genetic variation. Nucleic Acids Research. 2001;29(1):308–311. 10.1093/nar/29.1.308 11125122 PMC29783

[4] Frazer KA, Ballinger DG, Cox DR, Hinds DA, Stuve LL, Gibbs RA, et al A second generation human haplotype map of over 3.1 million SNPs. Nature. 2007;449(7164):851–861. 10.1038/nature06258 17943122 PMC2689609

[5] Salzman DW, Weidhaas JB. SNPing cancer in the bud: microRNA and microRNA-target site polymorphisms as diagnostic and prognostic biomarkers in cancer. Pharmacology & Therapeutics. 2013;137(1):55–63. 10.1016/j.pharmthera.2012.08.016 22964086 PMC3546232

[6] Moszyńska A, Gebert M, Collawn JF, Bartoszewski R. SNPs in microRNA target sites and their potential role in human disease. Open Biology. 2017;7(4):170019 10.1098/rsob.170019 28381629 PMC5413909

[7] Sethupathy P, Collins FS. MicroRNA target site polymorphisms and human disease. Trends in Genetics. 2008;24(10):489–497. 10.1016/j.tig.2008.07.004 18778868

[8] Nicoloso MS, Sun H, Spizzo R, Kim H, Wickramasinghe P, Shimizu M, et al Single-nucleotide polymorphisms inside microRNA target sites influence tumor susceptibility. Cancer Research. 2010;70(7):2789–2798. 10.1158/0008-5472.CAN-09-3541 20332227 PMC2853025

[9] Khan S, Greco D, Michailidou K, Milne RL, Muranen TA, Heikkinen T, et al MicroRNA related polymorphisms and breast cancer risk. PLoS One. 2014;9(11):e109973 10.1371/journal.pone.0109973 25390939 PMC4229095

[10] Yang H, Dinney CP, Ye Y, Zhu Y, Grossman HB, Wu X. Evaluation of genetic variants in microRNA-related genes and risk of bladder cancer. Cancer Research. 2008;68(7):2530–2537. 10.1158/0008-5472.CAN-07-5991 18381463

[11] Naccarati A, Pardini B, Stefano L, Landi D, Slyskova J, Novotny J, et al Polymorphisms in miRNA-binding sites of nucleotide excision repair genes and colorectal cancer risk. Carcinogenesis. 2012;33(7):1346–1351. 10.1093/carcin/bgs172 22581836

[12] Mullany LE, Wolff RK, Herrick JS, Buas MF, Slattery ML. SNP regulation of microRNA expression and subsequent colon cancer risk. PLoS One. 2015;10(12):e0143894 10.1371/journal.pone.0143894 26630397 PMC4667940

[13] Bhattacharya A, Ziebarth JD, Cui Y. PolymiRTS Database 3.0: linking polymorphisms in microRNAs and their target sites with human diseases and biological pathways. Nucleic Acids Research. 2013;42(D1):D86–D91. 10.1093/nar/gkt1028 24163105 PMC3965097

[14] Hiard S, Charlier C, Coppieters W, Georges M, Baurain D. Patrocles: a database of polymorphic miRNA-mediated gene regulation in vertebrates. Nucleic Acids Research. 2009;38(suppl_1):D640–D651. 10.1093/nar/gkp926 19906729 PMC2808989

[15] Hariharan M, Scaria V, Brahmachari SK. dbSMR: a novel resource of genome-wide SNPs affecting microRNA mediated regulation. BMC Bioinformatics. 2009;10(1):108 10.1186/1471-2105-10-108 19371411 PMC2676258

[16] Kumar S, Ambrosini G, Bucher P. SNP2TFBS–a database of regulatory SNPs affecting predicted transcription factor binding site affinity. Nucleic Acids Research. 2017;45(D1):D139–D144. 10.1093/nar/gkw1064 27899579 PMC5210548

[17] Richardson K, Lai CQ, Parnell LD, Lee YC, Ordovas JM. A genome-wide survey for SNPs altering microRNA seed sites identifies functional candidates in GWAS. BMC Genomics. 2011;12(1):504 10.1186/1471-2164-12-504 21995669 PMC3207998

[18] Wilkins OM, Titus AJ, Gui J, Eliot M, Butler RA, Sturgis EM, et al Genome-scale identification of microRNA-related SNPs associated with risk of head and neck squamous cell carcinoma. Carcinogenesis. 2017;. 10.1093/carcin/bgx056 28582492 PMC5862295

[19] Ryan BM, Robles AI, Harris CC. Genetic variation in microRNA networks: the implications for cancer research. Nature Reviews Cancer. 2010;10(6):389–402. 10.1038/nrc2867 20495573 PMC2950312

[20] Barenboim M, Zoltick BJ, Guo Y, Weinberger DR. MicroSNiPer: a web tool for prediction of SNP effects on putative microRNA targets. Human Mutation. 2010;31(11):1223–1232. 10.1002/humu.21349 20809528 PMC3001138

[21] Deveci M, Çatalyürek ÜV, Toland AE. mrSNP: Software to detect SNP effects on microRNA binding. BMC Bioinformatics. 2014;15(1):73 10.1186/1471-2105-15-73 24629096 PMC4067983

[22] Chi SW, Hannon GJ, Darnell RB. An Alternative Mode of microRNA Target Recognition. Nature Structural & Molecular Biology. 2012;19(3):321–327. 10.1038/nsmb.2230 PMC3541676 22343717

[23] Li Q, Seo JH, Stranger B, McKenna A, Pe’er I, LaFramboise T, et al Integrative eQTL-based analyses reveal the biology of breast cancer risk loci. Cell. 2013;152(3):633–641. 10.1016/j.cell.2012.12.034 23374354 PMC4165609

[24] Chen QR, Hu Y, Yan C, Buetow K, Meerzaman D. Systematic genetic analysis identifies Cis-eQTL target genes associated with glioblastoma patient survival. PLoS One. 2014;9(8):e105393 10.1371/journal.pone.0105393 25133526 PMC4136869

[25] Shpak M, Hall AW, Goldberg MM, Derryberry DZ, Ni Y, Iyer VR, et al An eQTL analysis of the human glioblastoma multiforme genome. Genomics. 2014;103(4):252–263. 10.1016/j.ygeno.2014.02.005 24607568

[26] Zhu Z, Zhang F, Hu H, Bakshi A, Robinson MR, Powell JE, et al Integration of summary data from GWAS and eQTL studies predicts complex trait gene targets. Nature Genetics. 2016;48(5):481–487. 10.1038/ng.3538 27019110

[27] Kristensen VN, Lingjærde OC, Russnes HG, Vollan HKM, Frigessi A, Børresen-Dale AL. Principles and methods of integrative genomic analyses in cancer. Nature Reviews Cancer. 2014;14(5):299–313. 10.1038/nrc3721 24759209

[28] Sun YV, Hu YJ. Integrative Analysis of Multi-omics Data for Discovery and Functional Studies of Complex Human Diseases; 2016. 10.1016/bs.adgen.2015.11.004 PMC5742494 26915271

[29] Wilk G, Braun R. Integrative analysis reveals disrupted pathways regulated by microRNAs in cancer. Nucleic Acids Research. 2018;46(3):1089–1101. 10.1093/nar/gkx1250 29294105 PMC5814839

[30] Tenenbaum JB, De Silva V, Langford JC. A global geometric framework for nonlinear dimensionality reduction. Science. 2000;290(5500):2319–2323. 10.1126/science.290.5500.2319 11125149

[31] Reich DE, Cargill M, Bolk S, Ireland J, Sabeti PC, Richter DJ, et al Linkage disequilibrium in the human genome. Nature. 2001;411(6834):199–204. 10.1038/35075590 11346797

[32] Benjamini Y, Hochberg Y. Controlling the false discovery rate: a practical and powerful approach to multiple testing. Journal of the Royal Statistical Society: Series B (Statistical Methodology). 1995; p. 289–300.

[33] Benjamini Y, Yekutieli D. The control of the false discovery rate in multiple testing under dependency. Annals of Statistics. 2001; p. 1165–1188.

[34] Clayton D. snpStats: SnpMatrix and XSnpMatrix classes and methods; 2015.

[35] Lewis BP, Shih Ih, Jones-Rhoades MW, Bartel DP, Burge CB. Prediction of mammalian microRNA targets. Cell. 2003;115(7):787–798. 10.1016/S0092-8674(03)01018-3 14697198

[36] Chou CH, Shrestha S, Yang CD, Chang NW, Lin YL, Liao KW, et al miRTarBase update 2018: a resource for experimentally validated microRNA-target interactions. Nucleic Acids Research. 2017;46(D1):D296–D302. 10.1093/nar/gkx1067 PMC5753222 29126174

[37] Betel D, Wilson M, Gabow A, Marks DS, Sander C. The microRNA.org resource: targets and expression. Nucleic Acids Research. 2008;36(Suppl 1):D149–D153. 10.1093/nar/gkm995 18158296 PMC2238905

[38] Vaňhara P, Horak P, Pils D, Anees M, Petz M, Gregor W, et al Loss of the oligosaccharyl transferase subunit TUSC3 promotes proliferation and migration of ovarian cancer cells. International Journal of Oncology. 2013;42(4):1383–1389. 10.3892/ijo.2013.1824 23404293

[39] Horak P, Tomasich E, Vaňhara P, Kratochvílová K, Anees M, Marhold M, et al TUSC3 loss alters the ER stress response and accelerates prostate cancer growth in vivo. Scientific Reports. 2014;4 10.1038/srep03739 24435307 PMC3894551

[40] Fan X, Zhang X, Shen J, Zhao H, Yu X, Chen Y, et al Decreased TUSC3 promotes pancreatic cancer proliferation, invasion and metastasis. PLoS One. 2016;11(2):e0149028 10.1371/journal.pone.0149028 26871953 PMC4752499

[41] Poola I, Abraham J, Marshalleck JJ, Yue Q, Fu SW, Viswanath L, et al Molecular constitution of breast but not other reproductive tissues is rich in growth promoting molecules: a possible link to highest incidence of tumor growths. FEBS Letters. 2009;583(18):3069–3075. 10.1016/j.febslet.2009.08.021 19698714 PMC2752361

[42] Cizeron-Clairac G, Lallemand F, Vacher S, Lidereau R, Bieche I, Callens C. MiR-190b, the highest up-regulated miRNA in ER*α*-positive compared to ER*α*-negative breast tumors, a new biomarker in breast cancers? BMC Cancer. 2015;15(1):1 10.1186/s12885-015-1505-5 26141719 PMC4491222

[43] Yu Y, Zhang D, Huang H, Li J, Zhang M, Wan Y, et al NF-*κ*B1 p50 promotes p53 protein translation through miR-190 downregulation of PHLPP1. Oncogene. 2014;33(8):996–1005. 10.1038/onc.2013.8 23396362 PMC3883870

[44] Stinson S, Lackner MR, Adai AT, Yu N, Kim HJ, O’Brien C, et al TRPS1 targeting by miR-221/222 promotes the epithelial-to-mesenchymal transition in breast cancer. Science Signaling. 2011;4(177):ra41–ra41. 10.1126/scisignal.2001538 21673316

[45] Turner N, Grose R. Fibroblast growth factor signalling: from development to cancer. Nature Reviews Cancer. 2010;10(2):116–129. 10.1038/nrc2780 20094046

[46] Li H, Yang L, Fu H, Yan J, Wang Y, Guo H, et al Association between G*α*i2 and ELMO1/Dock180 connects chemokine signalling with Rac activation and metastasis. Nature Communications. 2013;4:1706 10.1038/ncomms2680 23591873 PMC3644068

[47] Ho JY, Hsu RJ, Liu JM, Chen SC, Liao GS, Gao HW, et al MicroRNA-382-5p aggravates breast cancer progression by regulating the RERG/Ras/ERK signaling axis. Oncotarget. 2017;8(14):22443 10.18632/oncotarget.12338 27705918 PMC5410235

[48] Zhong S, Wyllie A, Barnes D, Wolf C, Spurr N. Relationship between the GSTM1 genetic polymorphism and susceptibility to bladder, breast and colon cancer. Carcinogenesis. 1993;14(9):1821–1824. 10.1093/carcin/14.9.1821 8403204

[49] Li D, Liu X, Lin L, Hou J, Li N, Wang C, et al MicroRNA-99a inhibits hepatocellular carcinoma growth and correlates with prognosis of patients with hepatocellular carcinoma. Journal of Biological Chemistry. 2011;286(42):36677–36685. 10.1074/jbc.M111.270561 21878637 PMC3196113

[50] Petrelli A, Perra A, Schernhuber K, Cargnelutti M, Salvi A, Migliore C, et al Sequential analysis of multistage hepatocarcinogenesis reveals that miR-100 and PLK1 dysregulation is an early event maintained along tumor progression. Oncogene. 2012;31(42):4517–4526. 10.1038/onc.2011.631 22249248

[51] Marshall L, White RJ. Non-coding RNA production by RNA polymerase III is implicated in cancer. Nature Reviews Cancer. 2008;8(12):911–914. 10.1038/nrc2539 18987635

[52] Yunlei Z, Zhe C, Yan L, Pengcheng W, Yanbo Z, Le S, et al INMAP, a novel truncated version of POLR3B, represses AP-1 and p53 transcriptional activity. Molecular and Cellular Biochemistry. 2013;374(1-2):81–89. 10.1007/s11010-012-1507-4 23124897

[53] Wang TH, Yeh CT, Ho JY, Ng KF, Chen TC. OncomiR miR-96 and miR-182 promote cell proliferation and invasion through targeting ephrinA5 in hepatocellular carcinoma. Molecular Carcinogenesis. 2016;55(4):366–375. 10.1002/mc.22286 25663355

[54] Wang J, Li J, Shen J, Wang C, Yang L, Zhang X. MicroRNA-182 downregulates metastasis suppressor 1 and contributes to metastasis of hepatocellular carcinoma. BMC Cancer. 2012;12(1):1 10.1186/1471-2407-12-227 22681717 PMC3492170

[55] Fanjul-Fernández M, Quesada V, Cabanillas R, Cadiñanos J, Fontanil T, Obaya Á, et al Cell–cell adhesion genes CTNNA2 and CTNNA3 are tumour suppressors frequently mutated in laryngeal carcinomas. Nature Communications. 2013;4 10.1038/ncomms3531 24100690

[56] McGranahan N, Favero F, de Bruin EC, Birkbak NJ, Szallasi Z, Swanton C. Clonal status of actionable driver events and the timing of mutational processes in cancer evolution. Science Translational Medicine. 2015;7(283):283ra54–283ra54. 10.1126/scitranslmed.aaa1408 25877892 PMC4636056

[57] Liu CJ, Shen WG, Peng SY, Cheng HW, Kao SY, Lin SC, et al miR-134 induces oncogenicity and metastasis in head and neck carcinoma through targeting WWOX gene. International Journal of Cancer. 2014;134(4):811–821. 10.1002/ijc.28358 23824713

[58] Sun CC, Li SJ, Li DJ. Hsa-miR-134 suppresses non-small cell lung cancer (NSCLC) development through down-regulation of CCND1. Oncotarget. 2016;7(24):35960 10.18632/oncotarget.8482 27166267 PMC5094975

[59] Hu WT, Yeh CC, Liu SY, Huang MC, Lai IR. The O-glycosylating enzyme GALNT2 suppresses the malignancy of gastric adenocarcinoma by reducing EGFR activities. American Journal of Cancer Research. 2018;8(9):1739 30323967 PMC6176175

[60] Ho WL, Chou CH, Jeng YM, Lu MY, Yang YL, Jou ST, et al GALNT2 suppresses malignant phenotypes through IGF-1 receptor and predicts favorable prognosis in neuroblastoma. Oncotarget. 2014;5(23):12247 10.18632/oncotarget.2627 25362349 PMC4322969

[61] Jia W, Eneh JO, Ratnaparkhe S, Altman MK, Murph MM. MicroRNA-30c-2* expressed in ovarian cancer cells suppresses growth factor induced cellular proliferation and downregulates the oncogene BCL9. Molecular Cancer Research. 2011; p. molcanres–0245. 10.1158/1541-7786.MCR-11-0245 22024689

[62] Shukla K, Sharma AK, Ward A, Will R, Hielscher T, Balwierz A, et al MicroRNA-30c-2-3p negatively regulates NF-*κ*B signaling and cell cycle progression through downregulation of TRADD and CCNE1 in breast cancer. Molecular Oncology. 2015;9(6):1106–1119. 10.1016/j.molonc.2015.01.008 25732226 PMC5528752

[63] Zöller M. CD44: can a cancer-initiating cell profit from an abundantly expressed molecule? Nature Reviews Cancer. 2011;11(4):254 10.1038/nrc3023 21390059

[64] Marhaba R, Zöller M. CD44 in cancer progression: adhesion, migration and growth regulation. Journal of Molecular Histology. 2004;35(3):211–231. 10.1023/B:HIJO.0000032354.94213.69 15339042

[65] Kinose Y, Sawada K, Nakamura K, Sawada I, Toda A, Nakatsuka E, et al The hypoxia-related microRNA miR-199a-3p displays tumor suppressor functions in ovarian carcinoma. Oncotarget. 2015;6(13):11342 10.18632/oncotarget.3604 25839163 PMC4484460

[66] Zhang W, Qian S, Yang G, Zhu L, Zhou B, Wang J, et al MicroRNA-199 suppresses cell proliferation, migration and invasion by downregulating RGS17 in hepatocellular carcinoma. Gene. 2018;659:22–28. 10.1016/j.gene.2018.03.053 29559347

[67] Zeng B, Shi W, Tan G. MiR-199a/b-3p inhibits gastric cancer cell proliferation via down-regulating PAK4/MEK/ERK signaling pathway. BMC Cancer. 2018;18(1):34 10.1186/s12885-017-3949-2 29304764 PMC5756398

[68] Kao CJ, Martiniez A, Shi XB, Yang J, Evans CP, Dobi A, et al miR-30 as a tumor suppressor connects EGF/Src signal to ERG and EMT. Oncogene. 2014;33(19):2495–2503. 10.1038/onc.2013.200 23728339 PMC4370229

[69] Qiu X, Dou Y. miR-1307 promotes the proliferation of prostate cancer by targeting FOXO3A. Biomedicine & Pharmacotherapy. 2017;88:430–435. 10.1016/j.biopha.2016.11.120 28122308

[70] Krasnov GS, Dmitriev AA, Melnikova NV, Zaretsky AR, Nasedkina TV, Zasedatelev AS, et al CrossHub: a tool for multi-way analysis of The Cancer Genome Atlas (TCGA) in the context of gene expression regulation mechanisms. Nucleic Acids Research. 2016; p. gkv1478. 10.1093/nar/gkv1478 26773058 PMC4838350

[71] Li Y, Liang M, Zhang Z. Regression analysis of combined gene expression regulation in acute myeloid leukemia. PLoS Computational Biology. 2014;10(10):e1003908 10.1371/journal.pcbi.1003908 25340776 PMC4207489

[72] Setty M, Helmy K, Khan AA, Silber J, Arvey A, Neezen F, et al Inferring transcriptional and microRNA-mediated regulatory programs in glioblastoma. Molecular Systems Biology. 2012;8(1):605 10.1038/msb.2012.37 22929615 PMC3435504

[73] Jacobsen A, Silber J, Harinath G, Huse JT, Schultz N, Sander C. Analysis of microRNA-target interactions across diverse cancer types. Nature Structural & Molecular Biology. 2013;20(11):1325–1332. 10.1038/nsmb.2678 PMC3982325 24096364

[74] Wang V, Wu W. MicroRNA-based therapeutics for cancer. BioDrugs. 2009;23(1):15–23. 10.2165/00063030-200923010-00002 19344188

[75] Kasinski AL, Kelnar K, Stahlhut C, Orellana E, Zhao J, Shimer E, et al A combinatorial microRNA therapeutics approach to suppressing non-small cell lung cancer. Oncogene. 2015;34(27):3547 10.1038/onc.2014.282 25174400 PMC4345154

[76] Shah MY, Ferrajoli A, Sood AK, Lopez-Berestein G, Calin GA. microRNA therapeutics in cancer—an emerging concept. EBioMedicine. 2016;12:34–42. 10.1016/j.ebiom.2016.09.017 27720213 PMC5078622

[77] Vogelstein B, Papadopoulos N, Velculescu VE, Zhou S, Diaz LA, Kinzler KW. Cancer genome landscapes. Science. 2013;339(6127):1546–1558. 10.1126/science.1235122 23539594 PMC3749880

