# Supplementary material for: regQTLs: Single nucleotide polymorphisms that modulate microRNA regulation of gene expression in tumors
Source: PLoS Genet. 2018 Dec 17;14(12):e1007837. doi: 10.1371/journal.pgen.1007837 (PMC6343932; doi:10.1371/journal.pgen.1007837)
Supplement: S1 Text — (PDF) [file pgen.1007837.s001.pdf]

Supplementary Information

regQTLs: Single Nucleotide Polymorphisms that Modulate microRNA Regulation of Gene Expression in Tumors

Gary Wilk and Rosemary Braun

1 Source code

Source code for this analysis is available from `https://github.com/gawilk/miRNA-SNP`

2 Full results tables

Tables of the results for all trios considered in the regQTL analysis of TCGA data may be accessed via the analysis git repository (`https://github.com/gawilk/miRNA-SNP`) or the Shiny App (`https://github.com/gawilk/mirApp`).

3 SNP PCA plots

We applied PCA to the SNP genotype data to adjust for population substructure using the snpStats package [1] in R. Most of the population substructure is explained by the first two principal components in each cancer type. Here we show the pairs plots for the first five principal components in each cancer type. The proportion of variance explained by the components can be seen in Fig. S-5.

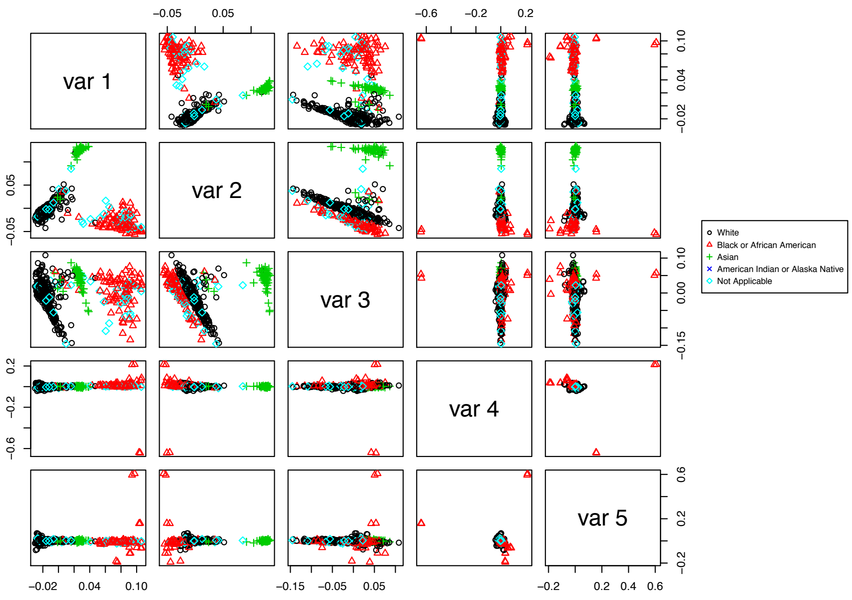

Figure S-1: PCA plot of Breast SNP genotype data. The population is stratified by ancestry/ethnicity, and substructure is largely explained by the first two principal components.

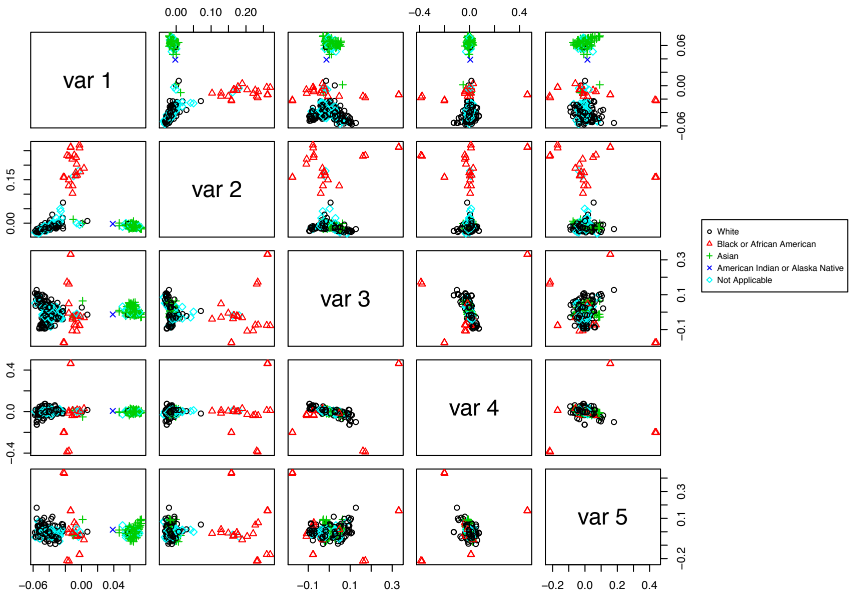

Figure S-2: PCA plot of Liver SNP genotype data.

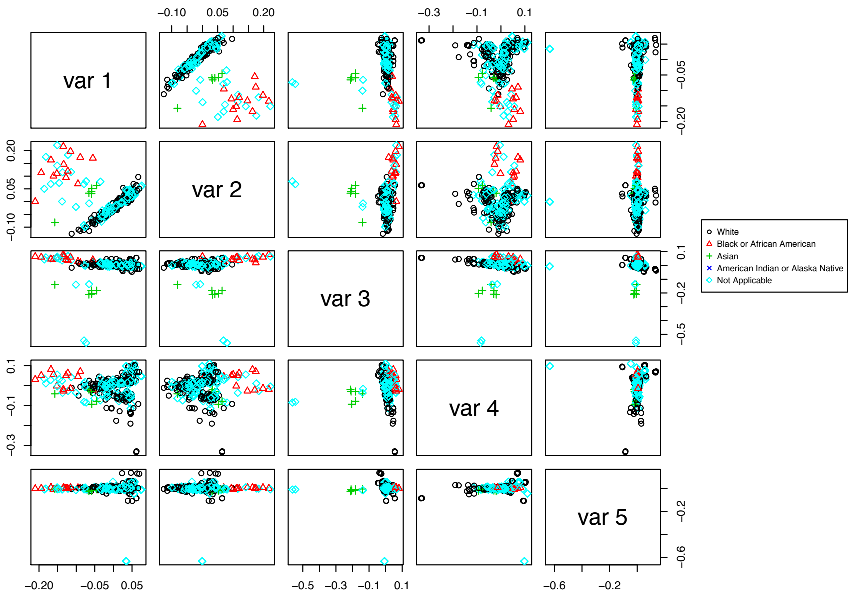

Figure S-3: PCA plot of Lung SNP genotype data.

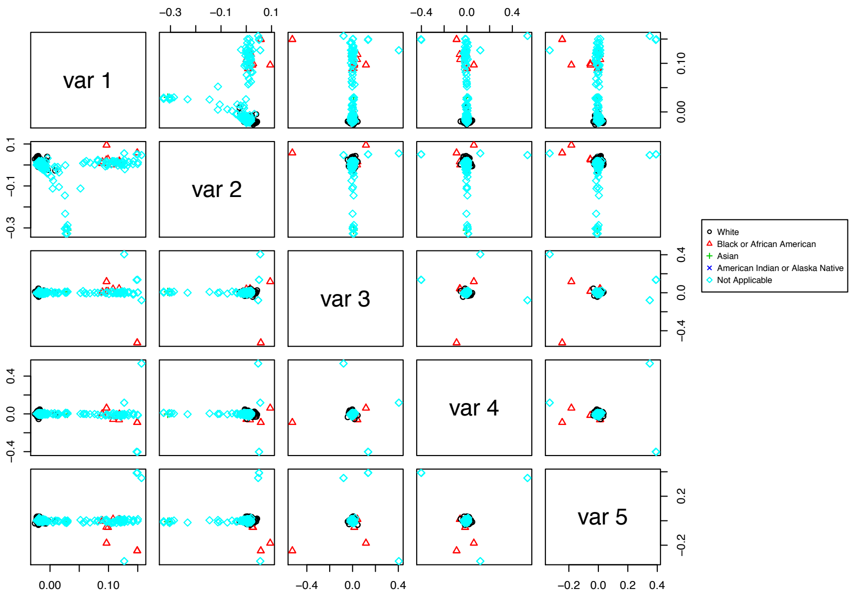

Figure S-4: PCA plot of Prostate SNP genotype data.

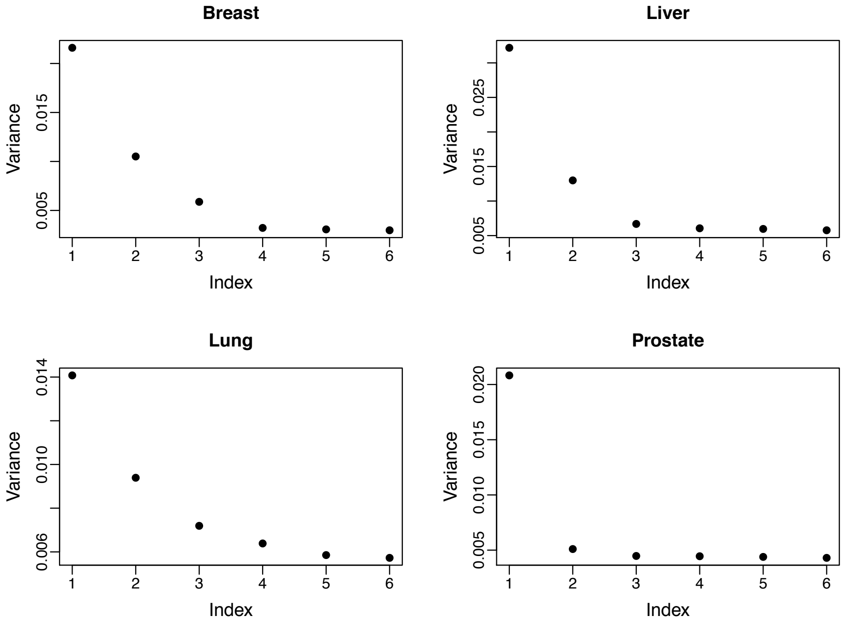

Figure S-5: Proportion of variance explained by the first six principal components.

4 SNP summary statistics

We consider SNPs from the TCGA Affymetrix SNP6.0 data that are in genes on the pathways that we previously found to exhibit disregulation. After filtering those SNPs according to our stated criteria (at minimum 1% minor allele frequency and 5% genotype frequency), we had 72901 unique SNPs in Breast Cancer, 70142 unique SNPs in Liver Cancer, 73037 unique SNPs in Lung Cancer, and 66967 unique SNPs in Prostate Cancer. As expected for tag SNPs, most SNPs lie in the intron regions of genes, shown in Table S-1.

Cancer type	3'UTR	5'UTR	intron	exon	coding
**Breast**	1218	248	49737	2468	886
**Liver**	1063	211	41625	2096	731
**Lung**	1130	231	45542	2277	806
**Prostate**	1075	221	43647	2171	771

Table S-1: Summary statistics for SNPs. Numbers denote unique SNPs within each genomic region per cancer type.

5 Breast tumor subtypes

Because breast cancer is a heterogeneous disease, we also under took a subtype analysis considering ER+, PR+, and triple negative breast cancer cases separately. Patients with ER+ and PR+ breast cancer largely overlap, as shown in the Venn diagram in Fig. S-6 with 438 common cases.

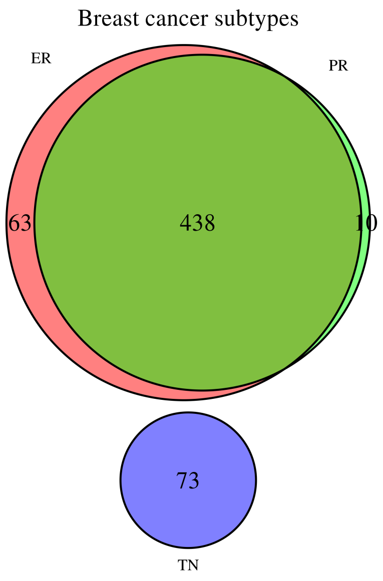

Figure S-6: Venn diagram of overlapping samples with ER+, PR+, and triple negative breast cancer.

In Fig. S-7 we illustrate qq-plots of regQTL p-values in ER+, PR+, and triple negative breast cancers. Each breast cancer subtype exhibits systematic deviations from the uniform distribution,

as does breast cancer in aggregate.

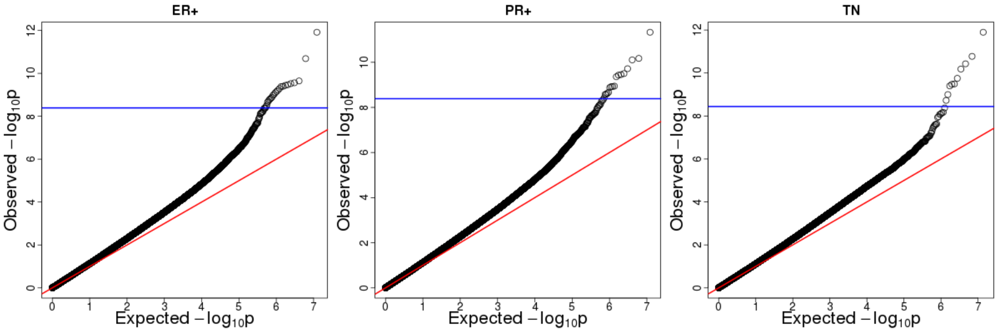

Figure S-7: Quantile-quantile plots of the observed p-values for the gene-miRNA-SNP ANOVA interaction tests versus their expected p-value distributions (the uniform distribution), tested in ER+, PR+, and triple negative breast cancer subtypes.

The complete results for the subtype analysis can be explored using the R Shiny app. In Fig. S-8 we show two example trio plots with ER+ and PR+ breast cancer. Triple negative breast cancer samples have an insufficient sample size to robustly compute regQTL interactions by genotype. In the left plot with ER+ samples, *TXNRD1* has been associated with poor outcomes in breast cancer patients and has been shown to be upregulated in *ERBB1* positive breast cancer tumors [2]. Its association with ER+ breast cancer in the literature gives some indication that our method is flagging relevant genes and mechanisms. Likewise, hsa-mir-3648 is upregulated in the presence of stress in the endoplasmic reticulum [3], and thereby downregulates *APC2* to promote cell proliferation. *APC2* is closely related to *APC*, a tumor suppressor protein, and exhibits similar tumor-suppressor effects in the Wnt pathway. In PR+ breast tumor samples (right plot), *CTNNA2* is strongly downregulated by hsa-mir-363 in the homozygous minor genotype. *CTNNA2* is tumor suppressor responsible for cell-cell adhesions that is frequently mutated in larygeal carcinoma [4]. Recently, hsa-mir-363 has been reported as a prognostic marker of liver cancer, with lower expression associated with better survival [5]. Additionally, hsa-mir-363 has been reported to behave as a tumor suppressor in gastric cancer [6]. Although *CTNNA2* and hsa-mir-363 are not directly implicated in breast cancer, these genes and miRNAs are implicated in other cancer related mechanisms in the literature.

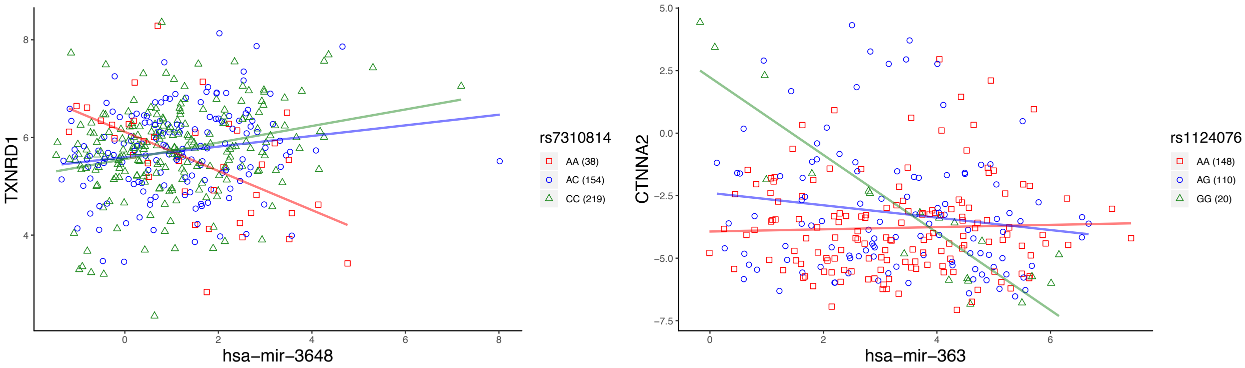

Figure S-8: Example trio plots in ER+ (left plot) and PR+ (right plot) breast cancer subtypes.

6 Significant regQTLs

In Table S-2, significant regQTLs and their associated statistics are shown. A large proportion of significant regQTL trios contain miRNA–mRNA relationships that are predicted by TargetScan [7]. However, only a small fraction of the regQTL trios in each cancer type have been experimentally validated for actual miRNA–mRNA regulatory relationships, according to mirTarBase [8]. Therefore, a substantial amount of regQTL trios require further experimental validation to verify their physiological effects. We note that TCGA miRNA names often report miRNA precursors and lack their mature miRNA variations; we therefore include mature miRNA targets under the precursor in predicted targets from TargetScan [7].

Cancer type	proportion	validated	total
**Breast**	0.58	51	2631
**Liver**	0.40	3	108
**Lung**	0.64	7	338
**Prostate**	0.66	5	209

Table S-2: Significant regQTLs (< 0.1 FDR) and their statistics. "Proportion" denotes the fraction of unique miRNA–mRNA relationships that are predicted by TargetScan [7], out of all unique miRNA–mRNA relationships found within the "total" number of significant regQTL trios. "Validated" denotes the unique number of experimentally validated miRNA–mRNA relationships according to mirTarBase [8], out of the significant regQTL trios.

There are several genes observed to contain more than one regQTL modulating their regulation by miRNAs, as displayed in Table S-3. In these cases, multiple regQTLs are in linkage disequilibrium and similarly affect gene regulation by genotype, and the genes themselves may be noteworthy candidates for futher investigation. Interestingly, only a few are known tumor suppressors and/or oncogenes from [9], whereas the rest perform other molecular functions.

Cancer type	genes	tumor suppressors
**Breast**	409	13
**Liver**	21	3
**Lung**	40	5
**Prostate**	42	1

Table S-3: Global review of significant regQTLs (at < 0.1 FDR). Genes denotes the number of genes containing > 1 SNP modulating miRNA–mRNA interactions, and the number of tumor suppressors and/or oncogenes according to [9] in each cancer type.

In addition, there are few genes and miRNAs that are common across all cancer types (Fig. S-9). The two genes are *STEAP3* and *HS3ST3A1*, and the nine miRNAs are hsa-let-7c, hsa-mir-100, hsa-mir-148a, hsa-mir-149, hsa-mir-370, hsa-mir-654, hsa-mir-758, hsa-mir-766, and hsa-mir-93. Both *STEAP3* and *HS3ST3A1* have been implicated in tumorigenic properties: *STEAP3* is part of the *STEAP* family of metalloreductases and has functions in exosome production, is part of the p53 network, and is thought to regulate tumor cell growth in hypoferratic conditions [10]. *HS3ST3A1* encodes for a sulfotransferase enzyme that has been recently implicated as a tumor regulator of breast cancer with tumor suppressor properties [11]. Both genes could be noteworthy candidates for further investigation.

Furthermore, the nine miRNAs in Fig. S-9 have been associated with cancer in the literature. hsa-mir-7c has been identified as a potential tumor suppressor that is frequently downregulated in prostate cancer [12], and has been recently identified as a miRNA that promotes tumor invasion in cholangiocarcinoma [13]. hsa-mir-100 downregulation has been implicated in liver cancer progression [14], and is a tumor suppressor targeting the mTOR pathway that is underexpressed in clear cell ovarian cancer [15]. hsa-mir-148a is thought to be an early prognostic marker in Hepatitis B mediated hepatocarcinoma [16], and is underexpressed in gastric cancer and associated with poor prognoses [17]. In addition, hsa-mir-149 is thought to behave as an oncogene and tumor supressor and is significantly dysregulated in several cancer types [18]. Another miRNA, hsa-mir-370, is thought to function as a tumor suppressor in laryngeal carcinoma and is downregulated in these tumors [19]. hsa-mir-654 has been implicated in oral squamous cell carcinoma as a potential biomarker due to its proliferative and metastatic potential [20]. In addition, hsa-mir-654 and other miRNAs mapped to its genomic locus, have been found to regulate proliferation, migration, and invasive properties in metastatic prostate cancer cells [21]. In liver cancer, hsa-mir-758 is reported to be a tumor suppressor and is underexpressed in liver cancer [22]. hsa-mir-766 is part of the p53 signaling pathway and has recently been proposed as a tumor suppressor by promoting p53 signaling, and is underexpressed in breast cancer [23]. In addition, repression of hsa-mir-766 through DNA methylation has been associated with aggresive renal cell carcinoma [24]. Finally, hsa-mir-93 is an oncogene that bolsters tumor growth and angiogenesis across several cancer types [25]. For instance, its upregulation in head and neck cancer is associated with metastasis and poor prognosis [26].

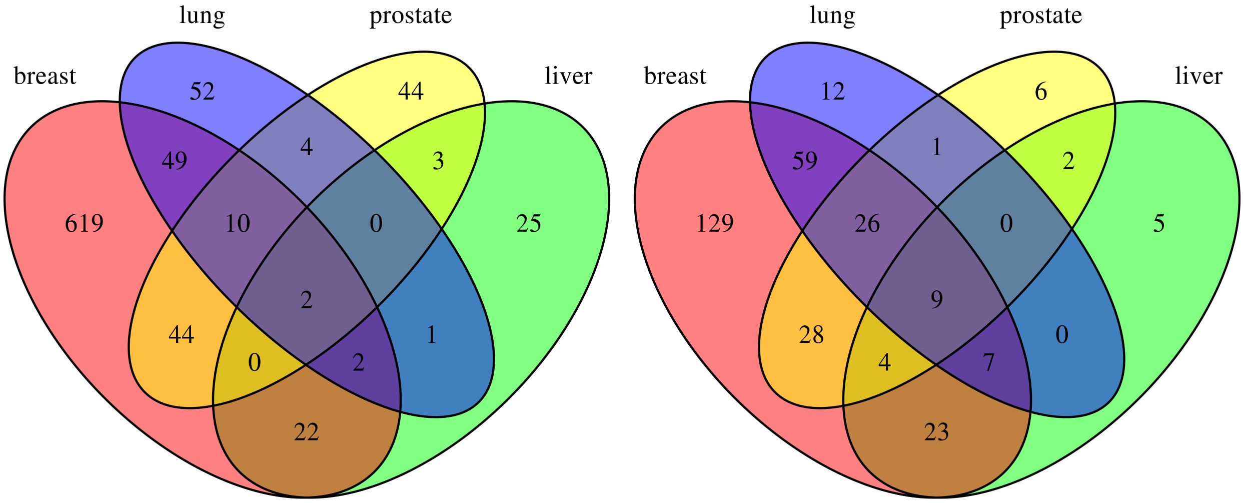

Figure S-9: Overlap of genes (left) and miRNAs (right) across cancer types in significant regQTL trios.

miRNA	gene	snps	total	MAF_{avg}	p_{MIN}	chr	predicted	pathways
hsa-mir-221	FGF14	13	309	4.88E-01	1.59E-03	13	TRUE	4
hsa-mir-141	EDA2R	10	34	4.93E-01	4.76E-02	X	TRUE	1
hsa-mir-141	FOXO1	10	38	4.74E-01	2.91E-02	13	TRUE	3
hsa-mir-642a	MTOR	10	36	4.60E-01	2.68E-02	1	FALSE	9
hsa-mir-125b-2	LRRC4C	9	437	4.85E-01	9.62E-03	11	FALSE	1
hsa-mir-222	FGF14	8	309	4.88E-01	7.10E-04	13	TRUE	4
hsa-mir-429	C7	7	53	5.15E-01	2.43E-02	5	FALSE	3
hsa-mir-642a	NUP210	7	164	5.16E-01	6.81E-02	3	FALSE	1
hsa-mir-24-2	TUSC3	7	218	4.96E-01	8.21E-03	8	TRUE	3
hsa-mir-134	CHSY3	6	67	4.72E-01	5.22E-02	5	FALSE	2
hsa-mir-218-2	COL11A1	6	115	4.89E-01	5.33E-02	1	TRUE	4
hsa-mir-3200	GSPT2	6	14	5.09E-01	5.92E-02	X	FALSE	1
hsa-mir-758	HTR1B	6	233	5.03E-01	3.24E-02	6	FALSE	1
hsa-let-7c	LRRC4C	6	437	4.85E-01	2.00E-03	11	FALSE	1
hsa-mir-190b	NUP210	6	164	5.16E-01	2.43E-02	3	FALSE	1

Table S-4: miRNA-gene pairs containing the greatest number of genetic variants significantly modulating their interactions in breast cancer. "SNPs" indicates the number of associated SNPs on the gene found to significantly modulate (at $p_{FDR} < 0.1$) the miRNA-gene interaction, out of the total number of known SNPs on the gene. MAF_{avg} indicates the average minor allele frequency of the SNPs located on the gene. p_{MIN} indicates the most significant interaction p-value after FDR-correction. "chr" indicates the chromosome where the SNP is located. "predicted" indicates whether the miRNA is predicted to target the gene based off sequence matching from microRNA.org. "pathways" indicates the number of KEGG pathways the gene is part of.

miRNA	gene	snps	total	MAF_{avg}	p_{MIN}	chr	predicted	pathways
hsa-mir-182	POLR3B	6	32	4.57E-01	9.01E-03	12	FALSE	5
hsa-mir-183	POLR3B	6	32	4.57E-01	1.91E-02	12	FALSE	5
hsa-mir-99a	STS	5	195	4.86E-01	4.20E-02	X	FALSE	1
hsa-mir-139	GLDC	3	49	5.35E-01	3.14E-02	9	FALSE	2
hsa-mir-107	NFYC	3	20	4.58E-01	2.07E-02	1	TRUE	1
hsa-mir-125b-1	STS	3	195	4.86E-01	2.15E-02	X	TRUE	1
hsa-mir-144	UGT2B7	3	23	5.18E-01	6.15E-02	4	FALSE	11
hsa-mir-122	ATP2B2	2	202	4.99E-01	3.28E-03	3	TRUE	3
hsa-mir-655	CACNA1C	2	262	4.64E-01	3.31E-02	12	TRUE	11
hsa-mir-215	CLDN1	2	59	5.07E-01	2.07E-02	3	FALSE	5
hsa-mir-215	DRD1	2	76	4.51E-01	6.81E-02	5	FALSE	3
hsa-mir-203	PLCE1	2	100	4.78E-01	3.17E-02	10	FALSE	4
hsa-mir-374b	RYR3	2	299	4.74E-01	2.07E-02	15	FALSE	3
hsa-mir-766	THOP1	2	3	2.75E-01	8.24E-02	19	TRUE	2
hsa-mir-34c	UGT1A6	2	35	4.82E-01	6.14E-02	2	FALSE	11

Table S-5: miRNA-gene pairs containing the greatest number of genetic variants significantly modulating their interactions in liver cancer.

miRNA	gene	snps	total	MAF_{avg}	p_{MIN}	chr	predicted	pathways
hsa-mir-411	COL11A1	7	107	4.72E-01	3.63E-02	1	FALSE	4
hsa-mir-766	MAOA	7	76	5.01E-01	3.70E-02	X	TRUE	8
hsa-mir-127	CTNNA2	6	614	5.04E-01	1.87E-03	2	FALSE	7
hsa-mir-134	CTNNA2	6	614	5.04E-01	1.87E-03	2	FALSE	7
hsa-mir-154	CTNNA2	6	614	5.04E-01	1.01E-02	2	TRUE	7
hsa-mir-369	CTNNA2	6	614	5.04E-01	4.78E-03	2	TRUE	7
hsa-mir-379	CTNNA2	6	614	5.04E-01	6.71E-03	2	FALSE	7
hsa-mir-409	CTNNA2	6	614	5.04E-01	1.87E-03	2	TRUE	7
hsa-mir-493	CTNNA2	6	614	5.04E-01	1.01E-02	2	FALSE	7
hsa-mir-496	CTNNA2	6	614	5.04E-01	1.28E-02	2	FALSE	7
hsa-mir-758	CTNNA2	6	614	5.04E-01	1.97E-02	2	FALSE	7
hsa-mir-379	COL11A1	5	107	4.72E-01	2.07E-02	1	FALSE	4
hsa-mir-370	CTNNA2	4	614	5.04E-01	1.87E-03	2	FALSE	7
hsa-mir-382	CTNNA2	4	614	5.04E-01	3.70E-02	2	FALSE	7
hsa-mir-200b	CTNNA3	4	567	4.94E-01	4.73E-02	10	FALSE	7

Table S-6: miRNA-gene pairs containing the greatest number of genetic variants significantly modulating their interactions in lung cancer.

miRNA	gene	snps	total	MAF_{avg}	p_{MIN}	chr	predicted	pathways
hsa-mir-26a-2	CNTN1	13	105	5.01E-01	8.25E-03	12	TRUE	1
hsa-mir-143	EFNA5	4	214	4.67E-01	3.14E-02	5	FALSE	1
hsa-mir-10a	FBP1	4	19	4.55E-01	8.25E-03	9	FALSE	5
hsa-mir-30a	FBXW7	4	24	5.49E-01	1.18E-02	4	TRUE	1
hsa-mir-421	WBSCR17	4	282	4.84E-01	2.19E-02	7	FALSE	0
hsa-mir-331	CACNA2D4	3	51	4.42E-01	6.05E-02	12	TRUE	5
hsa-mir-766	CACNA2D4	3	51	4.42E-01	3.63E-02	12	TRUE	5
hsa-mir-598	CDH3	3	23	5.39E-01	8.55E-02	16	TRUE	1
hsa-mir-1180	HS3ST3A1	3	172	5.05E-01	2.32E-02	17	FALSE	1
hsa-mir-330	MASP1	3	48	4.69E-01	2.19E-02	3	TRUE	2
hsa-mir-330	NEGR1	3	178	4.81E-01	7.91E-02	1	TRUE	1
hsa-mir-361	NTN4	3	40	5.09E-01	2.01E-02	12	TRUE	1
hsa-mir-15b	PARK2	3	576	4.97E-01	4.35E-02	6	TRUE	0
hsa-mir-1266	PDE4D	3	486	5.08E-01	9.82E-02	5	TRUE	1
hsa-mir-151	PRKCE	3	363	5.09E-01	4.30E-02	2	TRUE	5

Table S-7: miRNA-gene pairs containing the greatest number of genetic variants significantly modulating their interactions in prostate cancer.

References

[1] David Clayton. *snpStats: SnpMatrix and XSnpMatrix classes and methods*, 2015. R package version 1.24.0.

[2] Cristina Cadenas, Dennis Franckenstein, Marcus Schmidt, Mathias Gehrmann, Matthias Hermes, Bettina Geppert, Wiebke Schormann, Lindsey J Maccoux, Markus Schug, Anika Schumann, et al. Role of thioredoxin reductase 1 and thioredoxin interacting protein in prognosis of breast cancer. *Breast Cancer Research*, 12(3):R44, 2010.

[3] Farooq Rashid, Hassaan Mehboob Awan, Abdullah Shah, Liang Chen, and Ge Shan. Induction of mir-3648 upon er stress and its regulatory role in cell proliferation. *International Journal of Molecular Sciences*, 18(7):1375, 2017.

[4] Miriam Fanjul-Fernández, Víctor Quesada, Rubén Cabanillas, Juan Cadiñanos, Tania Fontanil, Álvaro Obaya, Andrew J Ramsay, José L Llorente, Aurora Astudillo, Santiago Cal, et al. Cell–cell adhesion genes ctnna2 and ctnna3 are tumour suppressors frequently mutated in laryngeal carcinomas. *Nature Communications*, 4, 2013.

[5] Jun Zhang, Jia Fan, Chongming Zhou, and Yanyu Qi. mir-363-5p as potential prognostic marker for hepatocellular carcinoma indicated by weighted co-expression network analysis of mirnas and mrna. *BMC Gastroenterology*, 17(1):81, 2017.

[6] Bo Song, Jin Yan, Chao Liu, Haiyang Zhou, and Yangchun Zheng. Tumor suppressor role of mir-363-3p in gastric cancer. *Medical Science Monitor: International Medical Journal of Experimental and Clinical Research*, 21:4074, 2015.

[7] Benjamin P Lewis, I-hung Shih, Matthew W Jones-Rhoades, David P Bartel, and Christopher B Burge. Prediction of mammalian microrna targets. *Cell*, 115(7):787–798, 2003.

[8] Chih-Hung Chou, Sirjana Shrestha, Chi-Dung Yang, Nai-Wen Chang, Yu-Ling Lin, Kuang-Wen Liao, Wei-Chi Huang, Ting-Hsuan Sun, Siang-Jyun Tu, Wei-Hsiang Lee, et al. mirtarbase update 2018: a resource for experimentally validated microrna-target interactions. *Nucleic Acids Research*, 46(D1):D296–D302, 2017.

[9] Bert Vogelstein, Nickolas Papadopoulos, Victor E Velculescu, Shibin Zhou, Luis A Diaz, and Kenneth W Kinzler. Cancer genome landscapes. *Science*, 339(6127):1546–1558, 2013.

[10] Taichi Isobe, Eishi Baba, Shuji Arita, Masato Komoda, Shingo Tamura, Tsuyoshi Shirakawa, Hiroshi Ariyama, Shigeo Takaishi, Hitoshi Kusaba, Takashi Ueki, et al. Human steap3 maintains tumor growth under hypoferric condition. *Experimental Cell Research*, 317(18):2582–2591, 2011.

[11] Xianqing Mao, Caroline Gauche, Michael WH Coughtrie, Catherine Bui, Sandrine Gulberti, Faten Merhi-Soussi, Nick Ramalanjaona, Isabelle Bertin-Jung, Alexandra Diot, Dominique Dumas, et al. The heparan sulfate sulfotransferase 3-ost3a (hs3st3a) is a novel tumor regulator and a prognostic marker in breast cancer. *Oncogene*, 35(38):5043, 2016.

[12] Nagalakshmi Nadiminty, Ramakumar Tummala, Wei Lou, Yezi Zhu, Xu-Bao Shi, June X Zou, Hongwu Chen, Jin Zhang, Xinbin Chen, Jun Luo, et al. Microrna let-7c is downregulated in prostate cancer and suppresses prostate cancer growth. *PloS One*, 7(3):e32832, 2012.

[13] Yu Xie, Hang Zhang, Xing-Jun Guo, Ye-Chen Feng, Rui-Zhi He, Xu Li, Shuo Yu, Yan Zhao, Ming Shen, Feng Zhu, et al. Let-7c inhibits cholangiocarcinoma growth but promotes tumor cell invasion and growth at extrahepatic sites. *Cell Death & Disease*, 9(2):249, 2018.

[14] A Petrelli, A Perra, K Schernhuber, M Cargnelutti, A Salvi, C Migliore, E Ghiso, A Benetti, S Barlati, GM Ledda-Columbano, et al. Sequential analysis of multistage hepatocarcinogenesis reveals that miR-100 and PLK1 dysregulation is an early event maintained along tumor progression. *Oncogene*, 31(42):4517–4526, 2012.

[15] Ankur K Nagaraja, Chad J Creighton, Zhifeng Yu, Huifeng Zhu, Preethi H Gunaratne, Jeffrey G Reid, Emuejevoke Olokpa, Hiroaki Itamochi, Naoto T Ueno, Shannon M Hawkins, et al. A link between mir-100 and frap1/mtor in clear cell ovarian cancer. *Molecular Endocrinology*, 24(2):447–463, 2010.

[16] Ke Yuan, Zhaorui Lian, Bill Sun, Marcia M Clayton, Irene OL Ng, and Mark A Feitelson. Role of mir-148a in hepatitis b associated hepatocellular carcinoma. *PloS One*, 7(4):e35331, 2012.

[17] Naoya Sakamoto, Yutaka Naito, Naohide Oue, Kazuhiro Sentani, Naohiro Uraoka, Htoo Zarni Oo, Kazuyoshi Yanagihara, Kazuhiko Aoyagi, Hiroki Sasaki, and Wataru Yasui. Microrna-148a is downregulated in gastric cancer, targets mmp7, and indicates tumor invasiveness and poor prognosis. *Cancer Science*, 105(2):236–243, 2014.

[18] Yunjie He, Dandan Yu, Lingping Zhu, Shanliang Zhong, Jianhua Zhao, and Jinhai Tang. mir-149 in human cancer: A systemic review. *Journal of Cancer*, 9(2):375, 2018.

[19] Wu Yungang, Li Xiaoyu, Taizhong Pang, Li Wenming, and Xinliang Pan. mir-370 targeted foxm1 functions as a tumor suppressor in laryngeal squamous cell carcinoma (lscc). *Biomedicine & Pharmacotherapy*, 68(2):149–154, 2014.

[20] Meng Lu, Chengyong Wang, Weihui Chen, Chuanqing Mao, and Jin Wang. mir-654-5p targets grap to promote proliferation, metastasis, and chemoresistance of oral squamous cell carcinoma through ras/mapk signaling. *DNA and Cell Biology*, 37(4):381–388, 2018.

[21] A Formosa, EK Markert, AM Lena, D Italiano, E Finazzi-Agro, AJ Levine, S Bernardini, AV Garabadgiu, G Melino, and E Candi. Micrornas, mir-154, mir-299-5p, mir-376a, mir-376c, mir-377, mir-381, mir-487b, mir-485-3p, mir-495 and mir-654-3p, mapped to the 14q32. 31 locus, regulate proliferation, apoptosis, migration and invasion in metastatic prostate cancer cells. *Oncogene*, 33(44):5173, 2014.

[22] Dan Jiang, William Cho, Zhenhao Li, Xiangrong Xu, Yuliang Qu, Zhongjia Jiang, Le Guo, and Guangxian Xu. Mir-758-3p suppresses proliferation, migration and invasion of hepatocellular carcinoma cells via targeting mdm2 and mtor. *Biomedicine & Pharmacotherapy*, 96:535–544, 2017.

[23] Qingqing Wang, Luke A Selth, and David F Callen. Mir-766 induces p53 accumulation and g2/m arrest by directly targeting mdm4. *Oncotarget*, 8(18):29914, 2017.

[24] Chen Chen, Sheng Xue, Jin Zhang, Wei Chen, Dongkui Gong, Jiayi Zheng, Junjie Ma, Wei Xue, Yonghui Chen, Wei Zhai, et al. Dna-methylation-mediated repression of mir-766-3p promotes cell proliferation via targeting sf2 expression in renal cell carcinoma. *International Journal of Cancer*, 141(9):1867–1878, 2017.

[25] L Fang, Z Deng, T Shatseva, J Yang, C Peng, WW Du, AJ Yee, LC Ang, C He, SW Shan, et al. Microrna mir-93 promotes tumor growth and angiogenesis by targeting integrin-β8. *Oncogene*, 30(7):806, 2011.

[26] Guo Li, Shuling Ren, Zhongwu Su, Chao Liu, Tengbo Deng, Donghai Huang, Yongquan Tian, Yuanzheng Qiu, and Yong Liu. Increased expression of mir-93 is associated with poor prognosis in head and neck squamous cell carcinoma. *Tumor Biology*, 36(5):3949–3956, 2015.
