# Supplementary material for: regQTLs: Single nucleotide polymorphisms that modulate microRNA regulation of gene expression in tumors
Source: PLoS Genet. 2018 Dec 17;14(12):e1007837. doi: 10.1371/journal.pgen.1007837 (PMC6343932; doi:10.1371/journal.pgen.1007837)
Supplement: S1 Table — Numbers denote unique SNPs within each genomic region per cancer type. (PDF) [file pgen.1007837.s002.pdf]

Cancer type	3'UTR	5'UTR	intron	exon	coding
**Breast**	1218	248	49737	2468	886
**Liver**	1063	211	41625	2096	731
**Lung**	1130	231	45542	2277	806
**Prostate**	1075	221	43647	2171	771
