# Supplementary material for: regQTLs: Single nucleotide polymorphisms that modulate microRNA regulation of gene expression in tumors
Source: PLoS Genet. 2018 Dec 17;14(12):e1007837. doi: 10.1371/journal.pgen.1007837 (PMC6343932; doi:10.1371/journal.pgen.1007837)
Supplement: S2 Table — “Proportion” denotes the fraction of unique miRNA–mRNA relationships that are predicted by TargetScan [35], out of all unique miRNA–mRNA relationships found within the “total” number of significant regQTL trios. “Validated” denotes the unique number of experimentally validated miRNA–mRNA relationships according to mirTarBase [36], out of the significant regQTL trios. (PDF) [file pgen.1007837.s003.pdf]

Cancer type	proportion	validated	total
**Breast**	0.58	51	2631
**Liver**	0.40	3	108
**Lung**	0.64	7	338
**Prostate**	0.66	5	209
