# Supplementary material for: regQTLs: Single nucleotide polymorphisms that modulate microRNA regulation of gene expression in tumors
Source: PLoS Genet. 2018 Dec 17;14(12):e1007837. doi: 10.1371/journal.pgen.1007837 (PMC6343932; doi:10.1371/journal.pgen.1007837)
Supplement: S3 Table — Genes denotes the number of genes containing > 1 SNP modulating miRNA–mRNA interactions, and the number of tumor suppressors and/or oncogenes according to [77] in each cancer type. (PDF) [file pgen.1007837.s004.pdf]

Cancer type	genes	tumor suppressors
**Breast**	409	13
**Liver**	21	3
**Lung**	40	5
**Prostate**	42	1
