# Supplementary material for: regQTLs: Single nucleotide polymorphisms that modulate microRNA regulation of gene expression in tumors
Source: PLoS Genet. 2018 Dec 17;14(12):e1007837. doi: 10.1371/journal.pgen.1007837 (PMC6343932; doi:10.1371/journal.pgen.1007837)
Supplement: S4 Table — “SNPs” indicates the number of associated SNPs on the gene found to significantly modulate (at pFDR < 0.1) the miRNA-gene interaction, out of the total number of known SNPs on the gene. MAFavg indicates the average minor allele frequency of the SNPs located on the gene. pMIN indicates the most significant interaction p-value after FDR-correction. “chr” indicates the chromosome where the SNP is located. “predicted” indicates whether the miRNA is predicted to target the gene based off sequence matching from microRNA.org. “pathways” indicates the number of KEGG pathways the gene is part of. (PDF) [file pgen.1007837.s005.pdf]

miRNA	gene	snps	total	MAF_{avg}	p_{MIN}	chr	predicted	pathways
hsa-mir-221	FGF14	13	309	4.88E-01	1.59E-03	13	TRUE	4
hsa-mir-141	EDA2R	10	34	4.93E-01	4.76E-02	X	TRUE	1
hsa-mir-141	FOXO1	10	38	4.74E-01	2.91E-02	13	TRUE	3
hsa-mir-642a	MTOR	10	36	4.60E-01	2.68E-02	1	FALSE	9
hsa-mir-125b-2	LRRC4C	9	437	4.85E-01	9.62E-03	11	FALSE	1
hsa-mir-222	FGF14	8	309	4.88E-01	7.10E-04	13	TRUE	4
hsa-mir-429	C7	7	53	5.15E-01	2.43E-02	5	FALSE	3
hsa-mir-642a	NUP210	7	164	5.16E-01	6.81E-02	3	FALSE	1
hsa-mir-24-2	TUSC3	7	218	4.96E-01	8.21E-03	8	TRUE	3
hsa-mir-134	CHSY3	6	67	4.72E-01	5.22E-02	5	FALSE	2
hsa-mir-218-2	COL11A1	6	115	4.89E-01	5.33E-02	1	TRUE	4
hsa-mir-3200	GSPT2	6	14	5.09E-01	5.92E-02	X	FALSE	1
hsa-mir-758	HTR1B	6	233	5.03E-01	3.24E-02	6	FALSE	1
hsa-let-7c	LRRC4C	6	437	4.85E-01	2.00E-03	11	FALSE	1
hsa-mir-190b	NUP210	6	164	5.16E-01	2.43E-02	3	FALSE	1
