# Supplementary material for: regQTLs: Single nucleotide polymorphisms that modulate microRNA regulation of gene expression in tumors
Source: PLoS Genet. 2018 Dec 17;14(12):e1007837. doi: 10.1371/journal.pgen.1007837 (PMC6343932; doi:10.1371/journal.pgen.1007837)
Supplement: S5 Table — (PDF) [file pgen.1007837.s006.pdf]

miRNA	gene	snps	total	MAF_{avg}	p_{MIN}	chr	predicted	pathways
hsa-mir-182	POLR3B	6	32	4.57E-01	9.01E-03	12	FALSE	5
hsa-mir-183	POLR3B	6	32	4.57E-01	1.91E-02	12	FALSE	5
hsa-mir-99a	STS	5	195	4.86E-01	4.20E-02	X	FALSE	1
hsa-mir-139	GLDC	3	49	5.35E-01	3.14E-02	9	FALSE	2
hsa-mir-107	NFYC	3	20	4.58E-01	2.07E-02	1	TRUE	1
hsa-mir-125b-1	STS	3	195	4.86E-01	2.15E-02	X	TRUE	1
hsa-mir-144	UGT2B7	3	23	5.18E-01	6.15E-02	4	FALSE	11
hsa-mir-122	ATP2B2	2	202	4.99E-01	3.28E-03	3	TRUE	3
hsa-mir-655	CACNA1C	2	262	4.64E-01	3.31E-02	12	TRUE	11
hsa-mir-215	CLDN1	2	59	5.07E-01	2.07E-02	3	FALSE	5
hsa-mir-215	DRD1	2	76	4.51E-01	6.81E-02	5	FALSE	3
hsa-mir-203	PLCE1	2	100	4.78E-01	3.17E-02	10	FALSE	4
hsa-mir-374b	RYR3	2	299	4.74E-01	2.07E-02	15	FALSE	3
hsa-mir-766	THOP1	2	3	2.75E-01	8.24E-02	19	TRUE	2
hsa-mir-34c	UGT1A6	2	35	4.82E-01	6.14E-02	2	FALSE	11
