# Supplementary material for: regQTLs: Single nucleotide polymorphisms that modulate microRNA regulation of gene expression in tumors
Source: PLoS Genet. 2018 Dec 17;14(12):e1007837. doi: 10.1371/journal.pgen.1007837 (PMC6343932; doi:10.1371/journal.pgen.1007837)
Supplement: S6 Table — (PDF) [file pgen.1007837.s007.pdf]

miRNA	gene	snps	total	MAF_{avg}	p_{MIN}	chr	predicted	pathways
hsa-mir-411	COL11A1	7	107	4.72E-01	3.63E-02	1	FALSE	4
hsa-mir-766	MAOA	7	76	5.01E-01	3.70E-02	X	TRUE	8
hsa-mir-127	CTNNA2	6	614	5.04E-01	1.87E-03	2	FALSE	7
hsa-mir-134	CTNNA2	6	614	5.04E-01	1.87E-03	2	FALSE	7
hsa-mir-154	CTNNA2	6	614	5.04E-01	1.01E-02	2	TRUE	7
hsa-mir-369	CTNNA2	6	614	5.04E-01	4.78E-03	2	TRUE	7
hsa-mir-379	CTNNA2	6	614	5.04E-01	6.71E-03	2	FALSE	7
hsa-mir-409	CTNNA2	6	614	5.04E-01	1.87E-03	2	TRUE	7
hsa-mir-493	CTNNA2	6	614	5.04E-01	1.01E-02	2	FALSE	7
hsa-mir-496	CTNNA2	6	614	5.04E-01	1.28E-02	2	FALSE	7
hsa-mir-758	CTNNA2	6	614	5.04E-01	1.97E-02	2	FALSE	7
hsa-mir-379	COL11A1	5	107	4.72E-01	2.07E-02	1	FALSE	4
hsa-mir-370	CTNNA2	4	614	5.04E-01	1.87E-03	2	FALSE	7
hsa-mir-382	CTNNA2	4	614	5.04E-01	3.70E-02	2	FALSE	7
hsa-mir-200b	CTNNA3	4	567	4.94E-01	4.73E-02	10	FALSE	7
