# Supplementary material for: regQTLs: Single nucleotide polymorphisms that modulate microRNA regulation of gene expression in tumors
Source: PLoS Genet. 2018 Dec 17;14(12):e1007837. doi: 10.1371/journal.pgen.1007837 (PMC6343932; doi:10.1371/journal.pgen.1007837)
Supplement: S7 Table — (PDF) [file pgen.1007837.s008.pdf]

miRNA	gene	snps	total	MAF_{avg}	p_{MIN}	chr	predicted	pathways
hsa-mir-26a-2	CNTN1	13	105	5.01E-01	8.25E-03	12	TRUE	1
hsa-mir-143	EFNA5	4	214	4.67E-01	3.14E-02	5	FALSE	1
hsa-mir-10a	FBP1	4	19	4.55E-01	8.25E-03	9	FALSE	5
hsa-mir-30a	FBXW7	4	24	5.49E-01	1.18E-02	4	TRUE	1
hsa-mir-421	WBSCR17	4	282	4.84E-01	2.19E-02	7	FALSE	0
hsa-mir-331	CACNA2D4	3	51	4.42E-01	6.05E-02	12	TRUE	5
hsa-mir-766	CACNA2D4	3	51	4.42E-01	3.63E-02	12	TRUE	5
hsa-mir-598	CDH3	3	23	5.39E-01	8.55E-02	16	TRUE	1
hsa-mir-1180	HS3ST3A1	3	172	5.05E-01	2.32E-02	17	FALSE	1
hsa-mir-330	MASP1	3	48	4.69E-01	2.19E-02	3	TRUE	2
hsa-mir-330	NEGR1	3	178	4.81E-01	7.91E-02	1	TRUE	1
hsa-mir-361	NTN4	3	40	5.09E-01	2.01E-02	12	TRUE	1
hsa-mir-15b	PARK2	3	576	4.97E-01	4.35E-02	6	TRUE	0
hsa-mir-1266	PDE4D	3	486	5.08E-01	9.82E-02	5	TRUE	1
hsa-mir-151	PRKCE	3	363	5.09E-01	4.30E-02	2	TRUE	5
