# Supplementary figures and images for: regQTLs: Single nucleotide polymorphisms that modulate microRNA regulation of gene expression in tumors
Source: PLoS Genet. 2018 Dec 17;14(12):e1007837. doi: 10.1371/journal.pgen.1007837 (PMC6343932; doi:10.1371/journal.pgen.1007837)

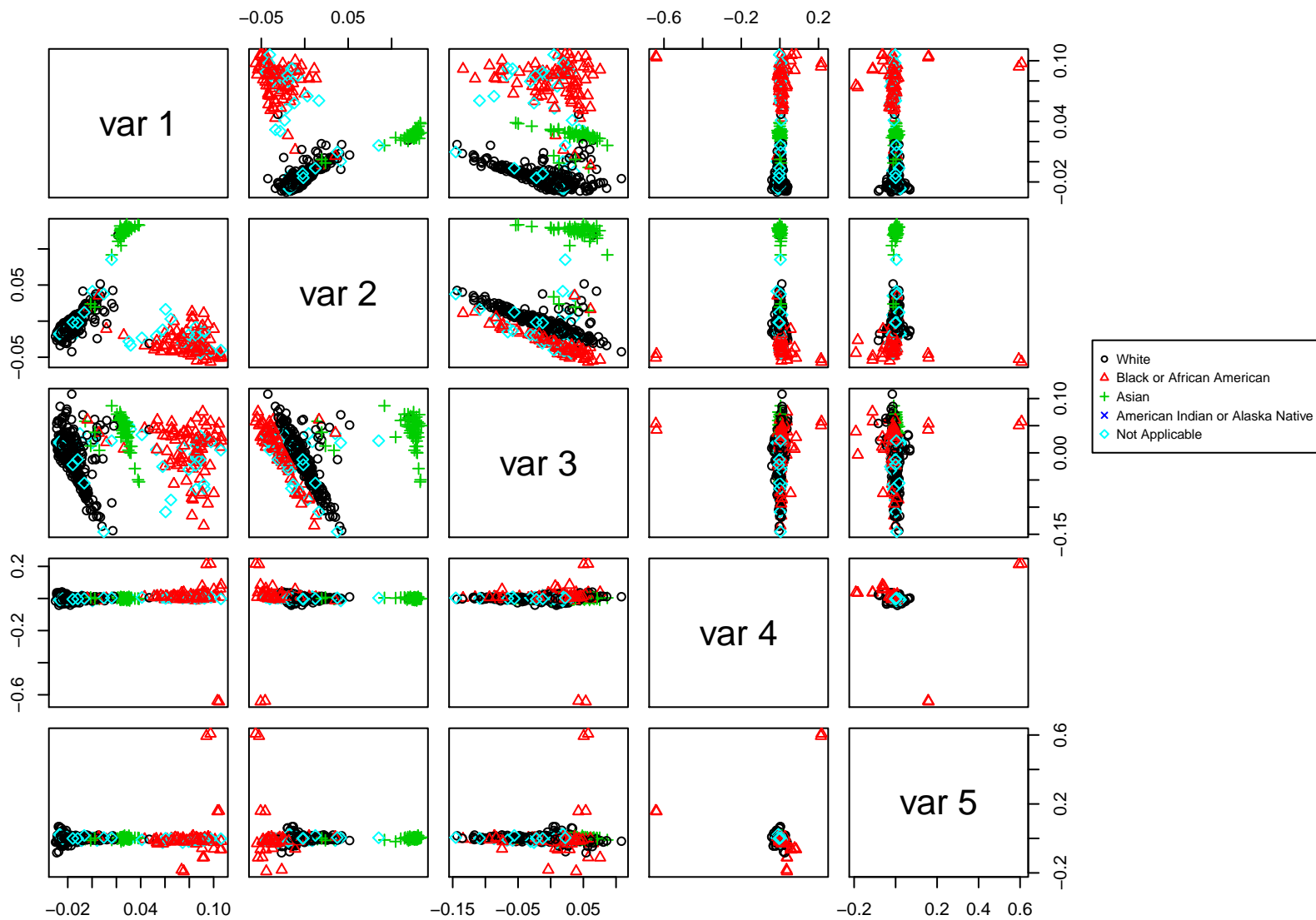

Supplement: S1 Fig — The population is stratified by ancestry/ethnicity, and substructure is largely explained by the first two principal components. (PDF) [file pgen.1007837.s009.pdf]

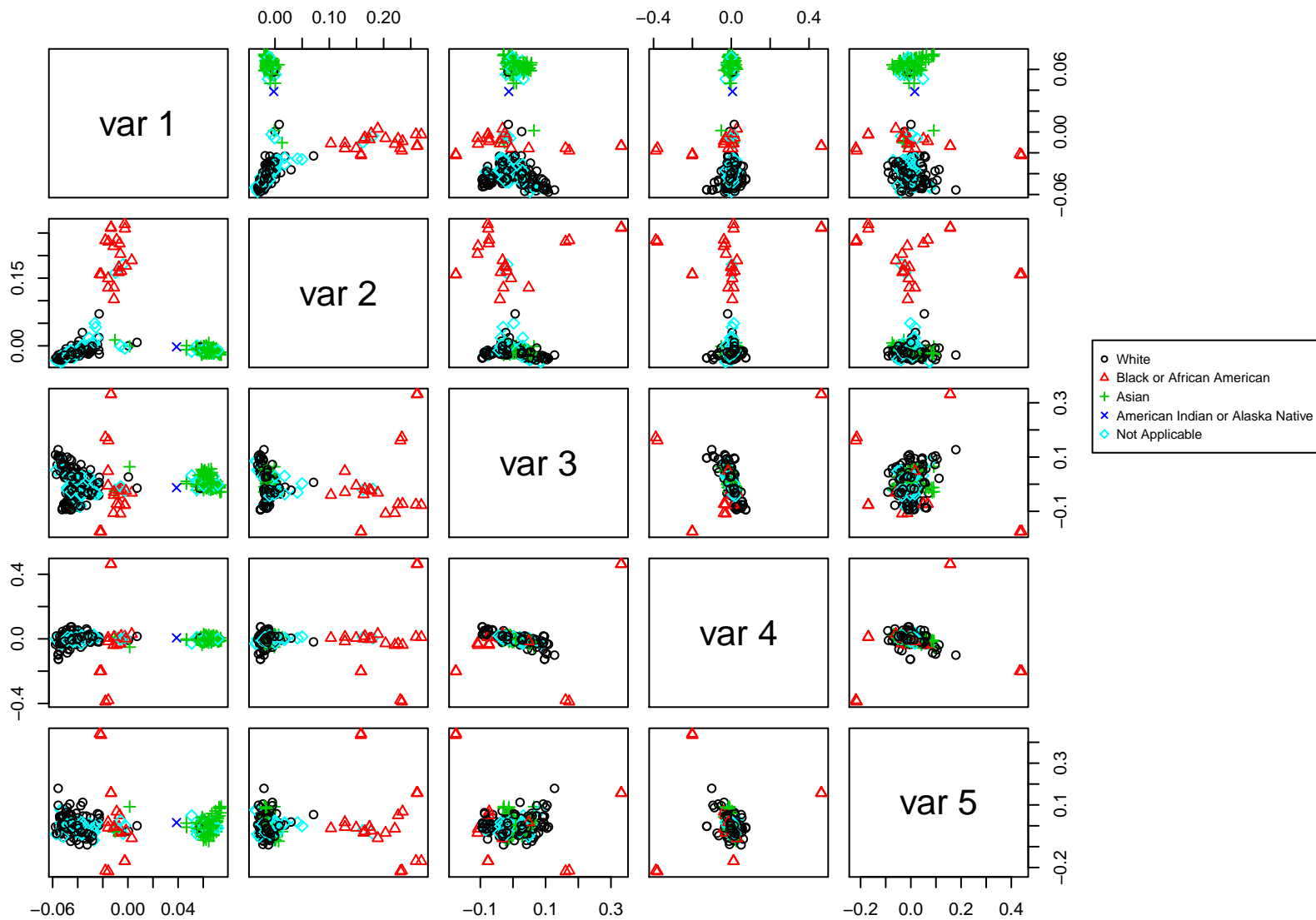

Supplement: S2 Fig — (PDF) [file pgen.1007837.s010.pdf]

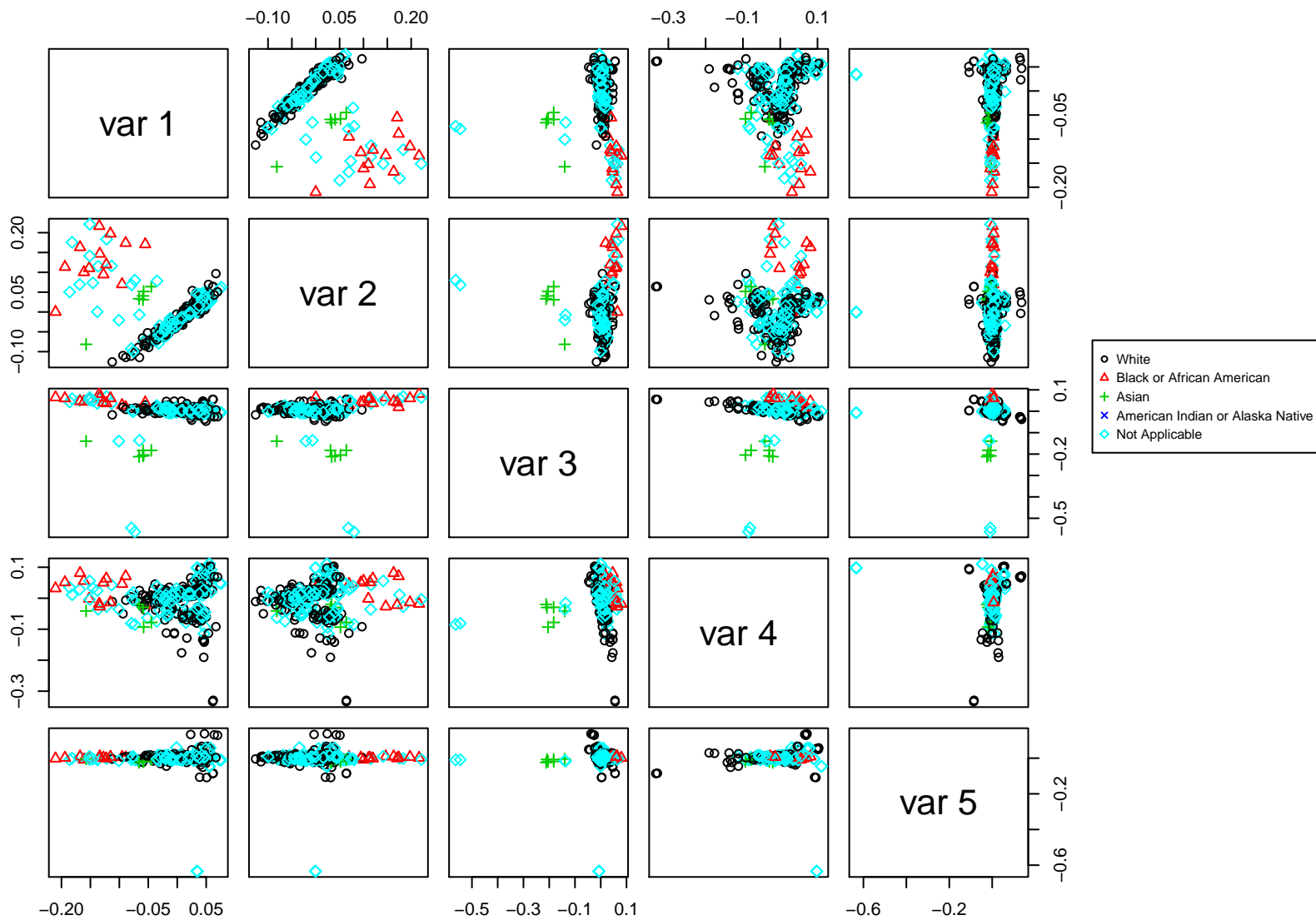

Supplement: S3 Fig — (PDF) [file pgen.1007837.s011.pdf]

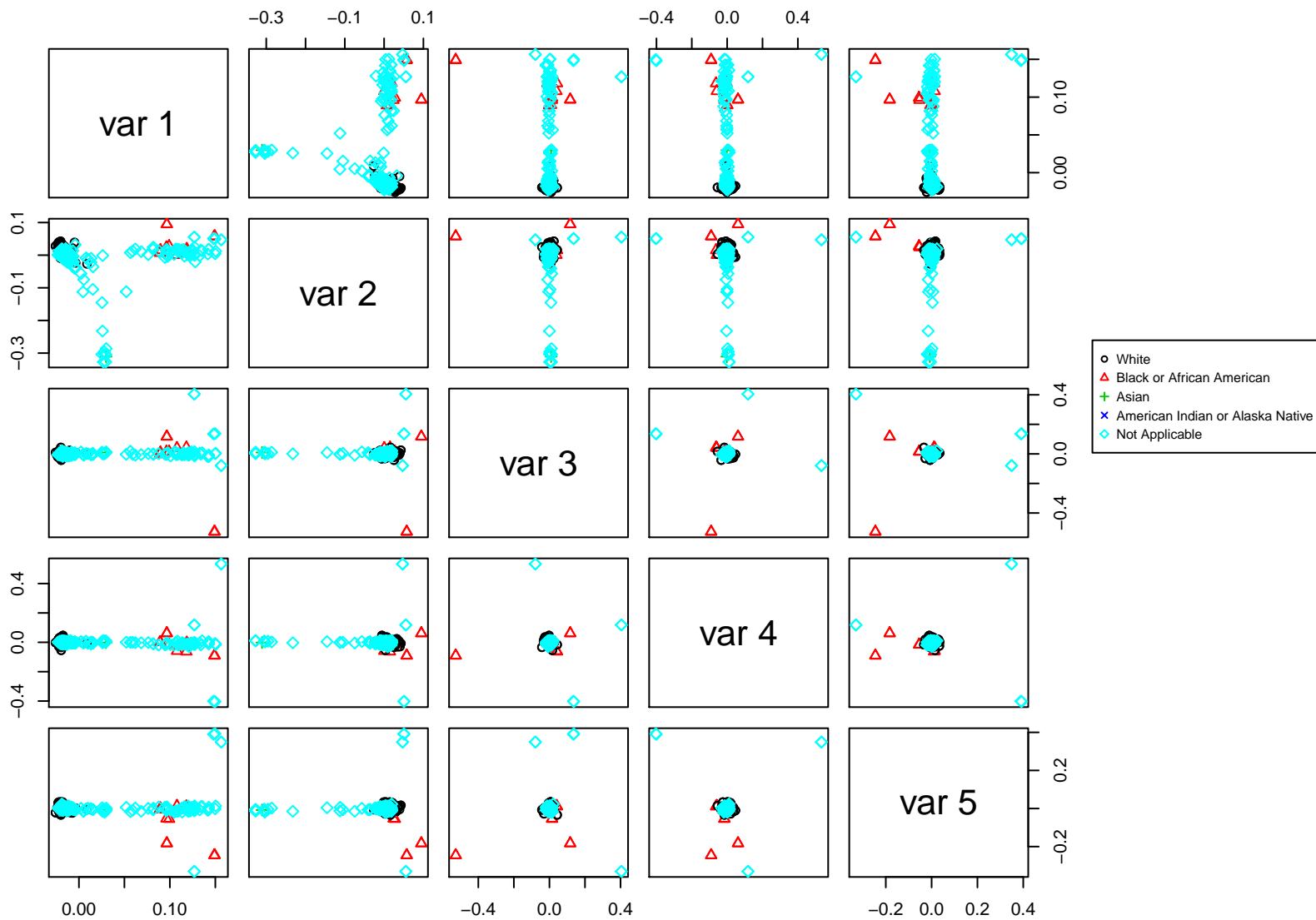

Supplement: S4 Fig — (PDF) [file pgen.1007837.s012.pdf]

**Breast**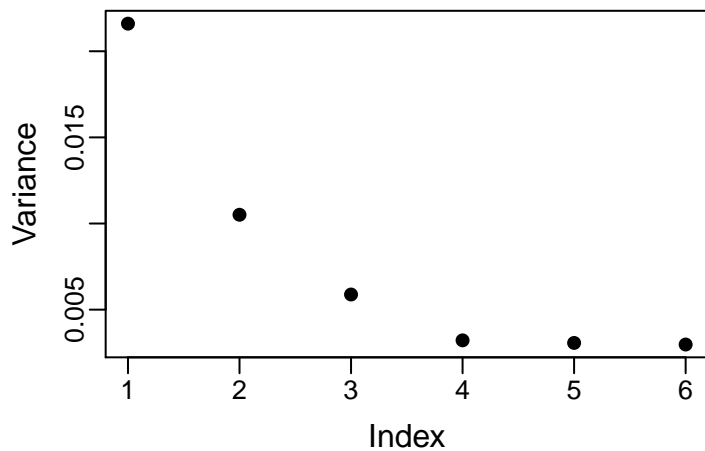**Liver**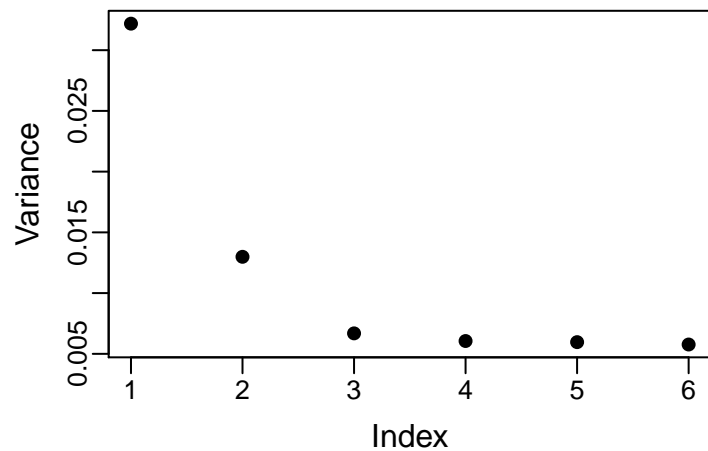**Lung**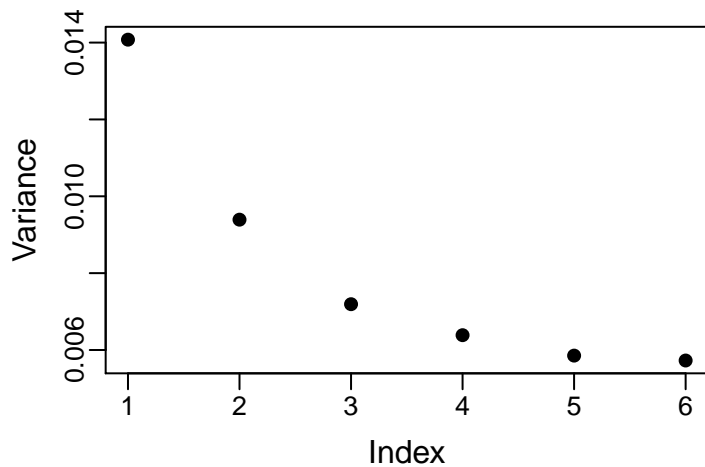**Prostate**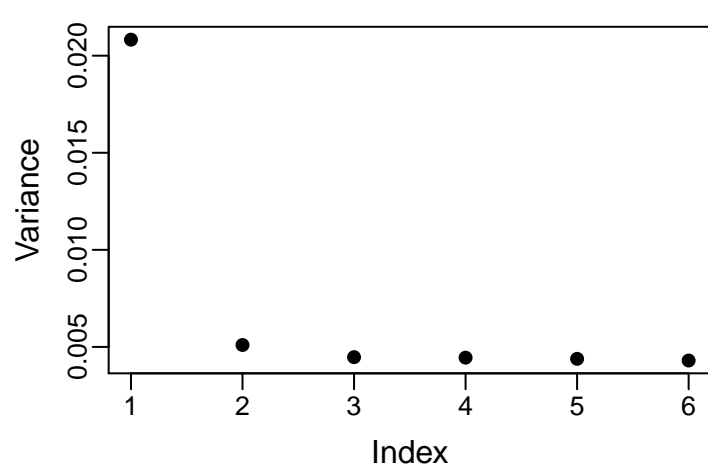

Supplement: S5 Fig — (PDF) [file pgen.1007837.s013.pdf]

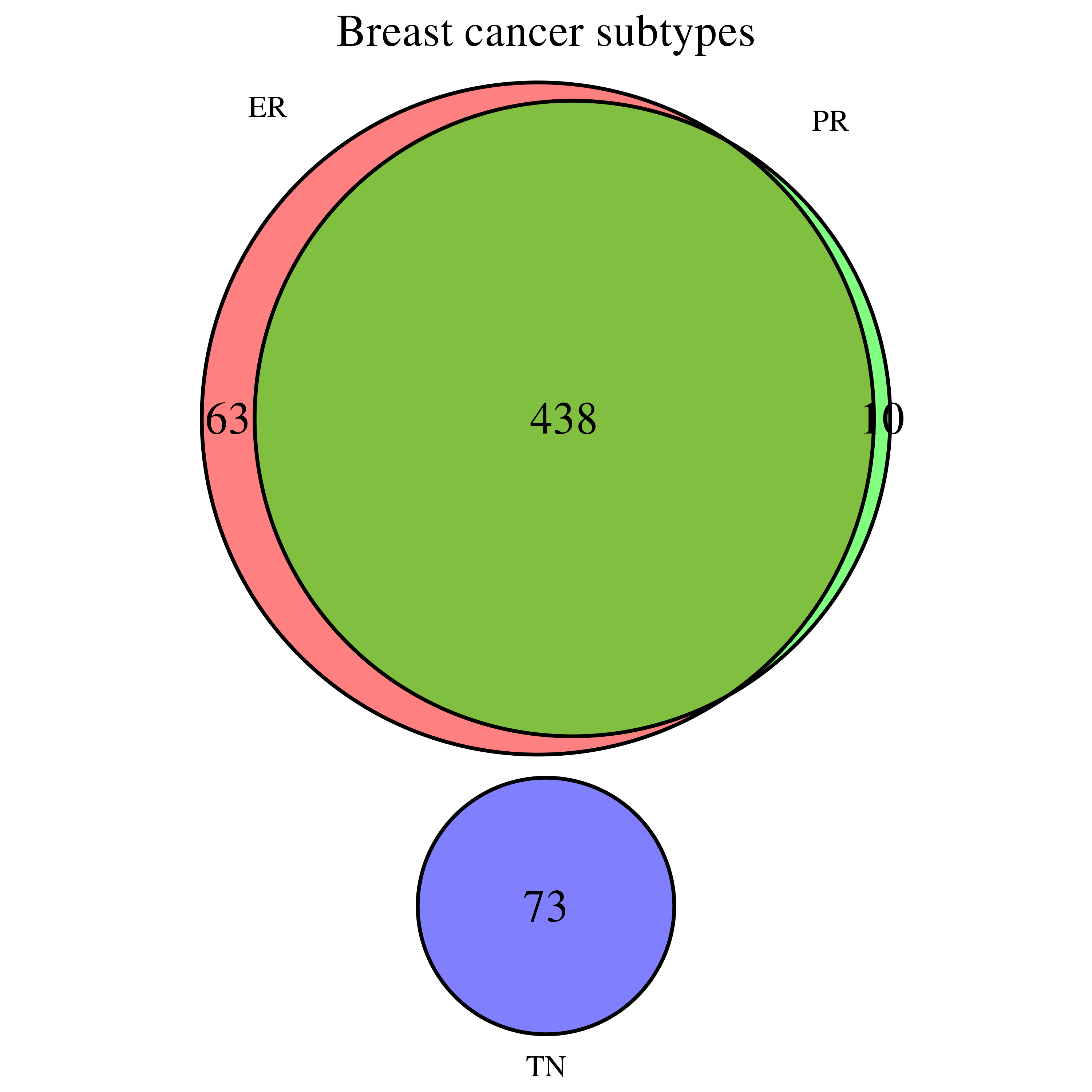

Supplement: S6 Fig — (PNG) [file pgen.1007837.s014.png]

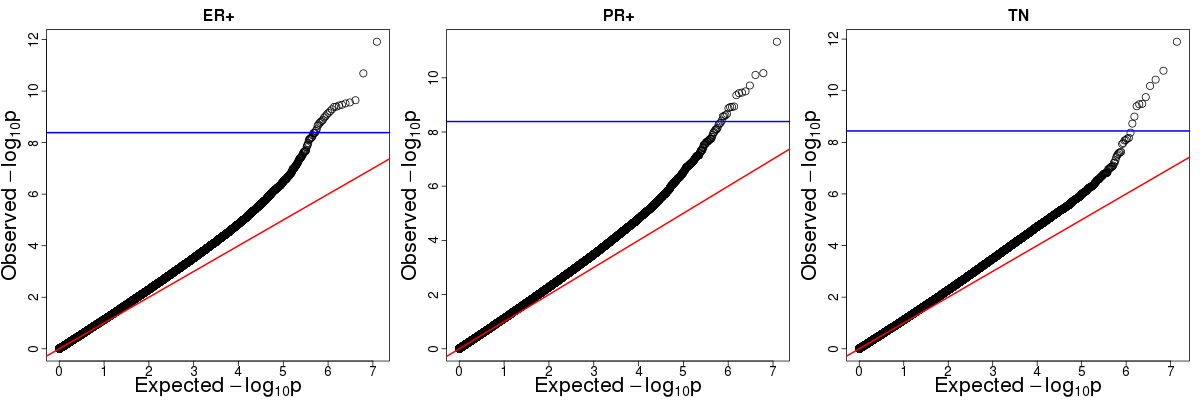

Supplement: S7 Fig — Quantile-quantile plots of the observed p-values for the gene-miRNA-SNP ANOVA interaction tests versus their expected p-value distributions (the uniform distribution), tested in ER+, PR+, and triple negative breast cancer subtypes. (PNG) [file pgen.1007837.s015.png]

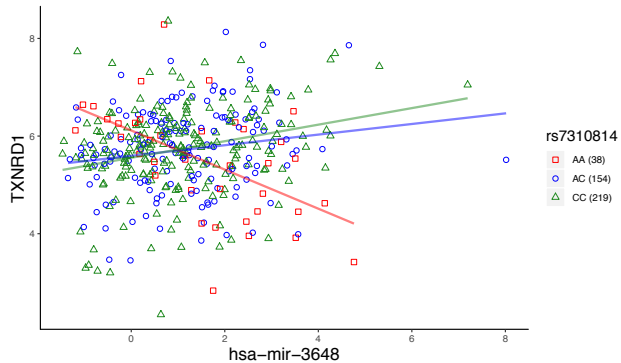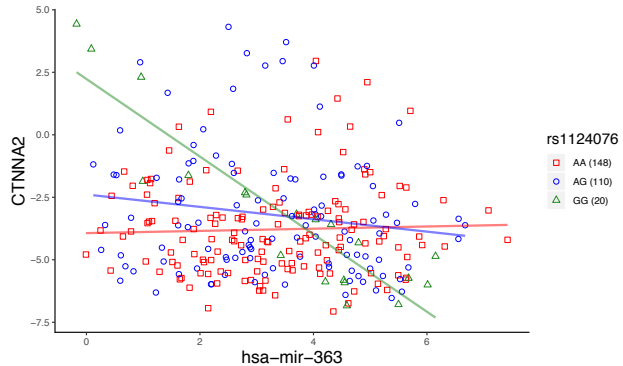

Supplement: S8 Fig — (PDF) [file pgen.1007837.s016.pdf]

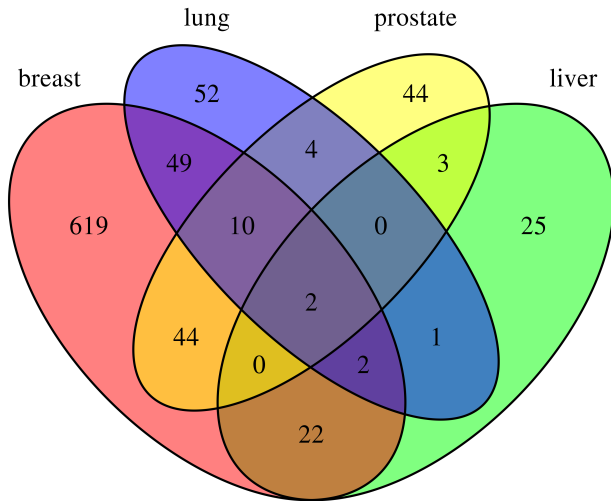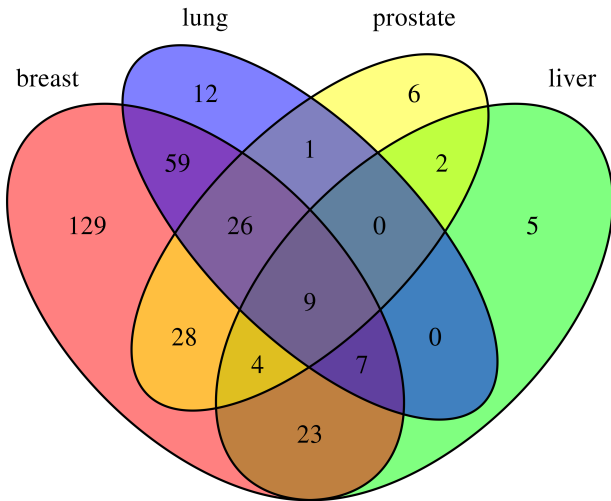

Supplement: S9 Fig — (PDF) [file pgen.1007837.s017.pdf]
